# Perceptions of values over time and why they matter

**DOI:** 10.1111/jopy.12608

**Published:** 2020-12-05

**Authors:** Colin M. G. Foad, Gregory G. R. Maio, Paul H. P. Hanel

**Affiliations:** ^1^ Department of Psychology Cardiff University Cardiff UK; ^2^ Department of Psychology University of Bath Bath UK; ^3^ Department of Psychology University of Essex Colchester UK

**Keywords:** behavioral intentions, value stability, value structure, values, values over time, well‐being

## Abstract

**Objective:**

Extant research mostly treats values as being stable over time. Our research examined how people perceive values over time and whether or not these perceptions reflect motivational tensions between theoretically opposing values. We also assessed the viability of examining values over time to predict well‐being and future intentions.

**Method:**

Four studies (*N* = 934) asked participants to report their values across past, present, and future settings. These temporal trajectories were analyzed across the four types of higher‐order values: self‐transcendence, self‐enhancement, openness, and conservation. Studies 3 and 4 assessed associations with well‐being. Study 4 assessed associations with self‐reported behavior over time.

**Results:**

Across all four studies, participants perceived their values as being dynamic over time. Younger participants' trajectories did not reflect the motivational conflicts typically reported in values research, but Study 4 showed potential awareness in older age groups. Variability in temporal values correlated with well‐being, particularly for openness values. Future values predicted future intentions, even when controlling for present values.

**Conclusion:**

This novel method of examining values provides new understanding into how people perceive the pursuit of values over time. Additionally, we show two ways that a temporal values measure can offer new insights into well‐being and future intentions.

## 1 INTRODUCTION

What did you used to care about most? What do you care most about today? And what do you think you will care about most in the future? We can learn a lot by asking ourselves these questions. McAdams' (2001) review outlined how evolving autobiographical narratives underpin the core of our identity. When we get the chance to reflect, key questions about our journey through life can tell us a lot about who we are and who we want to be. For example, did I really used to worry about that when I was younger (past)?; how can I get what matters to me now (present)?; and, where do I see my life heading (future)? It is clear that our motivations change over the course of our lives, either due to general maturation or experience of significant events. Furthermore, we can make estimates about how our priorities have changed and will change in the future.

While the literature on narrative identity intricately examines this component of change over time (e.g., Adler & McAdams, 2007), research on values has yet to address how people perceive their values over time. We thus wanted to explore whether people see their values as generally stable or dynamic across the lifespan, and how these trajectories vary for different value types. Perhaps more provocatively, we also wanted to test whether these perceptions map onto the motivational trade‐offs that have been consistently found to occur between values, (e.g., concern for the self and concern for others). When looking over time, we might fail to predict our values in a way that reflects conflicts between values we wish to pursue simultaneously—conflicts that we *do* recognize when constrained to view them in one time point (Schwartz et al., 2012). In addition to laying the foundations for understanding temporal value structures, we also wanted to explore how these value trajectories have theoretical utility for understanding well‐being and self‐reported behavior over time.

### 1.1 Value structure

Rokeach's (1973) seminal theory of values has been pivotal to understanding value structure and value change. According to Rokeach, value change is likely only when people perceive a conflict between their values and the self‐concept. Consistent with this perspective, previous research has tended to assume that values are essentially stable, particularly in the short‐term, but can vary to some degree when people are faced with new situations that force personal change and adaptation (Bardi et al., 2009; Rudnev, 2014). For instance, in a longitudinal study, it was found that students shifted away from extrinsic values over their college career (Sheldon, 2005). The challenges of aging may also explain why and how values change, with age correlating positively with conservation values and negatively with openness values (Caprara et al., 2003; Milfont et al., 2016; Robinson, 2013). In sum, any temporal changes in value importance are likely to occur either because of a change in the person's external environment, or because of a significant change in their internal motivation.

**FIGURE 1:**
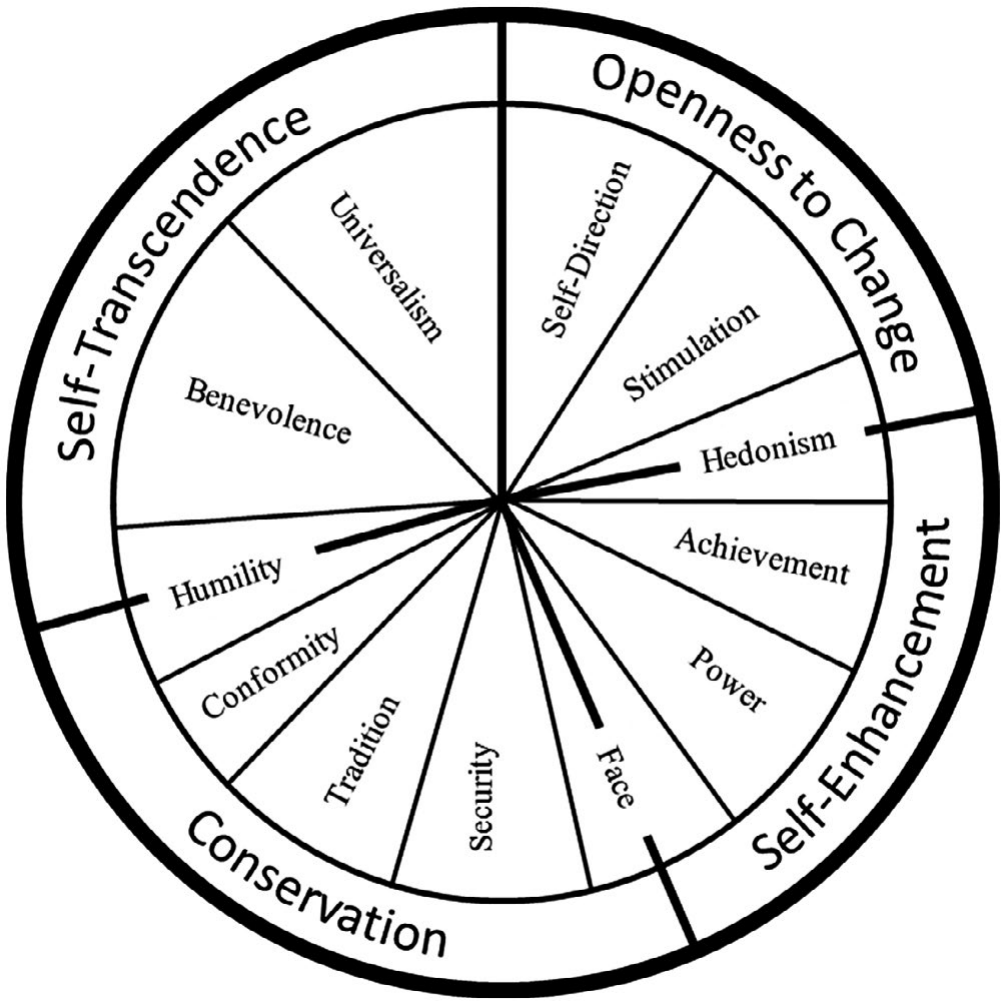
Circular model of values, adapted from Schwartz et al. (2012)

But the perception of value change and the directionality of these perceptions is an interesting open question. Very little research has considered whether people see their own values as changing over time and none has examined if such changes occur in particular directions. Schwartz's (1992) influential cross‐cultural model of values helps to illustrate the importance of this issue. According to the theory, values express motives that can be mapped in a circular model. In this circular structure, competing motivations appear opposite one another and complementary motivations appear close together (see Figure 1). The precise number, definition, and structure of each value in the model has varied from its initial foundation (Schwartz & Bilsky, 1987) to more recent revisions (Schwartz et al., 2012). However, the broader dimensions from self‐transcendence to self‐enhancement and from openness to conservation have remained essentially the same. Furthermore, the validity of the circular structure is reflected by evidence of tensions across the circle. For example, people who attach more importance to self‐transcendence values also attach less importance to self‐enhancement values (Schwartz & Sagiv, 1995).

The model has been highly influential (see Maio, 2016) because of empirical support in over 80 nations around the world (Schwartz et al., 2012), and the values included within the model help to predict a range of social attitudes and are sensitive to the surrounding social context (Boer & Fischer, 2013). For example, individuals who were higher in openness values and lower in conservation values were more ready to embrace out‐group contact (Sagiv & Schwartz, 1995). However, the most important evidence supporting the model directly examines its assumptions about the circular pattern of motivational relations between values. The structure has a wealth of supporting evidence from a range of samples and contexts (see Schwartz et al., 2012), across cross‐sectional (Bardi & Schwartz, 2003; Bilsky et al., 2011), experimental (e.g., Maio, Pakizeh, et al., 2009; Pakizeh et al., 2007), and neuroimaging paradigms (e.g., Brosch et al., 2011; Leszkowicz et al., 2017).

Perhaps most pertinent to our research, the model helps to predict patterns of value change: when values have been shown to change over time, the pattern of change shows that values at opposite ends of the circle change in opposing directions and, consequently, the value structure is maintained (Bardi et al., 2009; Vecchione et al., 2016). Thus, the circular structure is found across a range of situations and remains valid even when people change the importance they attach to different values.

A final important point to note is that two main methods of analyzing values data exist (see Borg & Bardi, 2016). First, as Schwartz (2009) generally recommends, researchers can look at mean‐centered (or ipsatized) data. There are two main advantages to this format that are relevant to our research. One advantage is that it allows for comparison of individuals while controlling for variation in their use of the response scale. The other advantage is that it allows researchers to assess which values have changed in relative importance. The second method of measurement (see He et al., 2017; He & van de Vijver, 2015), is to look at the raw scores. The main advantage of this format is that it more likely reflects a direct representation of a participant's values and avoids the pitfalls of removing potentially meaningful variance from the mean rating (Borg & Bardi, 2016). For our purposes it is important to look at both formats, as we are introducing a novel method of assessing values within‐persons, which could impact upon both approaches. It is plausible that participants could both predict raw changes in value importance over time, as well as shifting relative value priorities within each time point.

### 1.2 Value stability and change over time

A fundamental question is whether people perceive tensions between theoretically opposing values (e.g., wealth and equality) when they consider their own values over time. Important consequences emanate from how people perceive their motivations as changing from past to present to future. Some perceived trajectories can differ substantially from actual temporal change, but they remain relevant to attitudes and behavior. For example, individuals who place greater emphasis on considering future consequences are more likely to exercise and eat healthily (Dassen et al., 2016; Joireman et al., 2012). Similarly, past and potential selves are independent predictors of future task performance (Elliot et al., 2015). In addition, self‐evaluations differ when the future is projected as close or distant, but in varying directions (Heller et al., 2011; Wilson et al., 2012). Some evidence indicates that there is greater positivity toward close future selves compared to distant future selves (Wilson et al., 2012). Other research, however, indicates that there is greater positivity toward self‐concepts placed in the distant future, compared to the near future (Heller et al., 2011). While there are substantial and meaningful differences between the theoretical foundations of the self‐concept and values, such evidence does suggest a broader need to continue to refine our understanding of self‐perceptions over time.

Interestingly, no research has yet assessed the role of different values perceived in the present as a contrast to values in the past or future. The closest methodological match is one study in Quoidbach, Gilbert, and Wilson's work (2013) where they asked participants to report how much they thought they had changed ten basic values from 10 years earlier, or to predict the change in these values in 10 years' time (no participants assessed past, present, and future simultaneously). Their “End of History Illusion,” also found in personality traits, showed that people reported comparatively more change from their past than they predicted change into their future. However, because only absolute changes between past‐present or present‐future were analyzed, this research offers an interesting overview of how people view aspects of their selves as temporally fluid, but it does not provide detail on how those values interact over time, the direction of change, nor how such motivations may be necessarily in competition with one another.

### 1.3 Values and well‐being

People's perceptions of values over time may be linked to their well‐being. Psychological well‐being is a multifaceted construct with three key components: life satisfaction, affective well‐being, and psychological flourishing (Diener et al., 2010). Life satisfaction can be measured using the Satisfaction with Life Scale (SWLS), which taps into cognitive judgments of well‐being (Diener et al., 1985). Affective well‐being can be measured using the Scale of Positive and Negative Experience (SPANE), which identifies the current strength of emotional states (Diener et al., 2010). Psychological flourishing can be measured with the Flourishing Scale (FS), which captures the extent to which people feel they are functioning well in important areas of human life (Diener et al., 2010). All three components are considered in our exploration of values and well‐being.

Some researchers consider personal values to be relevant to every aspect of well‐being, because values necessarily interact with the subjective experiences that contribute to each individual's happiness (Felce & Perry, 1995). That is, people receive joy from different things, and these differences can be captured by knowing their values and their social context. Consistent with this link, Sagiv and Schwartz (2000) found business students to have a positive relationship between power values and well‐being, while the relationship was negative for psychology students. Similarly, students who considered achievement values to be highly important reported greater well‐being when they experienced success in terms of academic performance, while students who strongly valued hedonistic values were happier on days they had gone to a party (Oishi et al., 1999). In both these studies, the sizes of the relations were mostly small (*r* < .25). Research has also highlighted the impact of broader socio‐political settings upon the values‐well‐being relationship, with more individualistic settings increasing the role of the openness‐conservation dimension, while attenuating the role of the self‐transcendence‐self‐enhancement dimension (Sortheix & Schwartz, 2017). In sum, to understand the link between values and well‐being, it is important to know the value orientations of the individual, relevant details of the social context, and to consider different components of well‐being (Burr et al., 2011).

In the field of narrative identity, links between well‐being and temporal perspective have been found, but it is noted there is a paucity of research assessing how unity and coherence of self‐identity relate to well‐being (Adler et al., 2016). While it is important to note that the theory and methodology of studying self‐identity and values are substantively different, it does offer a plausible foundation for finding associations between perceptions of values over time and well‐being. For our purposes, the relationship between perceived value trajectories over time and psychological well‐being was an interesting new issue. Stability in values over time may reflect a current contentment with past and predicted selves, or a perceived lack of progress from the past and openness for change in the future. As an exploratory avenue, we therefore wished to ascertain whether greater perceived stability in value trajectories also predicted well‐being in general, and whether this impact depended on other factors such as the individual's age and the dimension of well‐being being evaluated.

### 1.4 Present research

In four studies, we explored perceptions of values over time by asking participants to rate the importance of their values in the past, present, and future. Many procedures force participants to consider a specific point in the past or the future (e.g., Quoidbach et al., 2013; Ryff, 1991; Wilson & Ross, 2001). For the first three studies, we chose to avoid specifying exact time points. Specific time points make people mindful of situational pressures of *that* specific time. For instance, students may think of specific dates in the recent past that possess specific contextual factors (e.g., graduation, exams). By deliberately eliciting a more general perspective, we enabled participants’ abstract motives and beliefs to influence their reported value trajectories. However, to test how our novel method compares to comparable procedures that do specify time points, we included this approach in Study 4.

We had two primary and four secondary aims across the studies. The first primary aim was to test whether people see their values as stable or dynamic (i.e., are there significant changes in value importance across the past, present, and future, or do people assign similar levels of importance to each value at each time point?). The second primary aim was to test whether theoretical models of values that presuppose opposition between values were supported by people's own forecasted progress. The secondary aims were to test if asking people to report their values over time would impact upon value importance in the current context (i.e., compared to a standard measure); to test how stability of values related to current well‐being; to test if future values could predict behavioral intentions; to test whether perceived value trajectories were moderated by age.

Based on related previous research (Quoidbach et al., 2013), we assumed a small‐to‐medium effect size (*f* = .175) between past, present, and future values (repeated measures ANOVA). A power analysis using GPower 3.1.9.4 revealed that to detect such an effect with a power of .95 a sample size of at least 86 is required (assuming a correlation of .50 between past, present, and future values). Thus, we aimed to recruit at least 86 participants per study.

## 2 STUDY 1

Our first study tested whether there are particular directions of perceived value change over time and whether these perceptions align with Schwartz's model. To enhance the possibility that participants would perceive potential opposition between values over time, we only presented participants with values that oppose one another in the model (i.e., self‐transcendence vs. self‐enhancement, or openness vs. conservation). One potentially important moderating variable, the Preference for Consistency (PFC) scale (Cialdini et al., 1995), was included at the end of the design. There were no reliable effects of this measure, and therefore, it is not considered further in this report.^1^

### 2.1 Method

#### 2.1.1 Participants

Participants were 124 undergraduate students at a British university (117 women, 7 men) who took part for course credit. They were between 18 and 28 years of age (*M* = 21). All participants completed the study in the laboratory.

#### 2.1.2 Design

A mixed design was used. All participants completed an adapted version of the Schwartz (1992) Values Scale (SVS), which assessed the importance attached to values over time.^2^ Participants were randomly assigned to complete a version that examined the self‐transcendence and self‐enhancement dimension or a version that examined the openness and conservation dimension. Time (past, present, and future) was the within‐participant factor.

#### 2.1.3 Procedure

Participants completed the values measurement using pen and paper in individual laboratories. After completing the task, participants were debriefed and thanked.

### 2.2 Materials

An adapted version of the (SVS) was used to measure how participants saw their values as changing over time (see Appendix A for an example of the layout used). By placing the time points side‐by‐side and in chronological order, the task encouraged participants to draw a trajectory over time for each value. Participants either received 10 values representing the self‐transcendence (helpfulness, responsibility, forgiveness, equality, and honesty) *and* self‐enhancement (power, wealth, success, ambition, and influence) values, or they received 10 values representing the openness (creativity, adventure, curiosity, an exciting life, and a varied life) *and* conservation (politeness, moderate tendency, respect for tradition, obedience, and devotion) values. For each value, participants were asked to rate the importance of each value as a guiding principle in their life in the past, present, and future. Answers were provided on a scale from −1 (*opposed to my values*) to 7 (*of supreme importance*).

To provide value scores for each motivational domain, the five values in each domain were averaged at each time point separately (past, current, and future). Cronbach's *α* varied from .69 to .77 for self‐transcendence values, .62 to .71 for self‐enhancement values, .51 to .61 for openness values, and .56 to .59 for conservation values. Although some of these *α* coefficients were low (Lance et al., 2006), they were comparable with other research using shortened versions of the SVS (e.g., Lindeman & Verkasalo, 2005), particularly given the breadth of value type that each score necessarily encompasses and the long‐established validity of the measure (Sortheix & Lönnqvist, 2014).

### 2.3 Results and discussion

**FIGURE 2:**
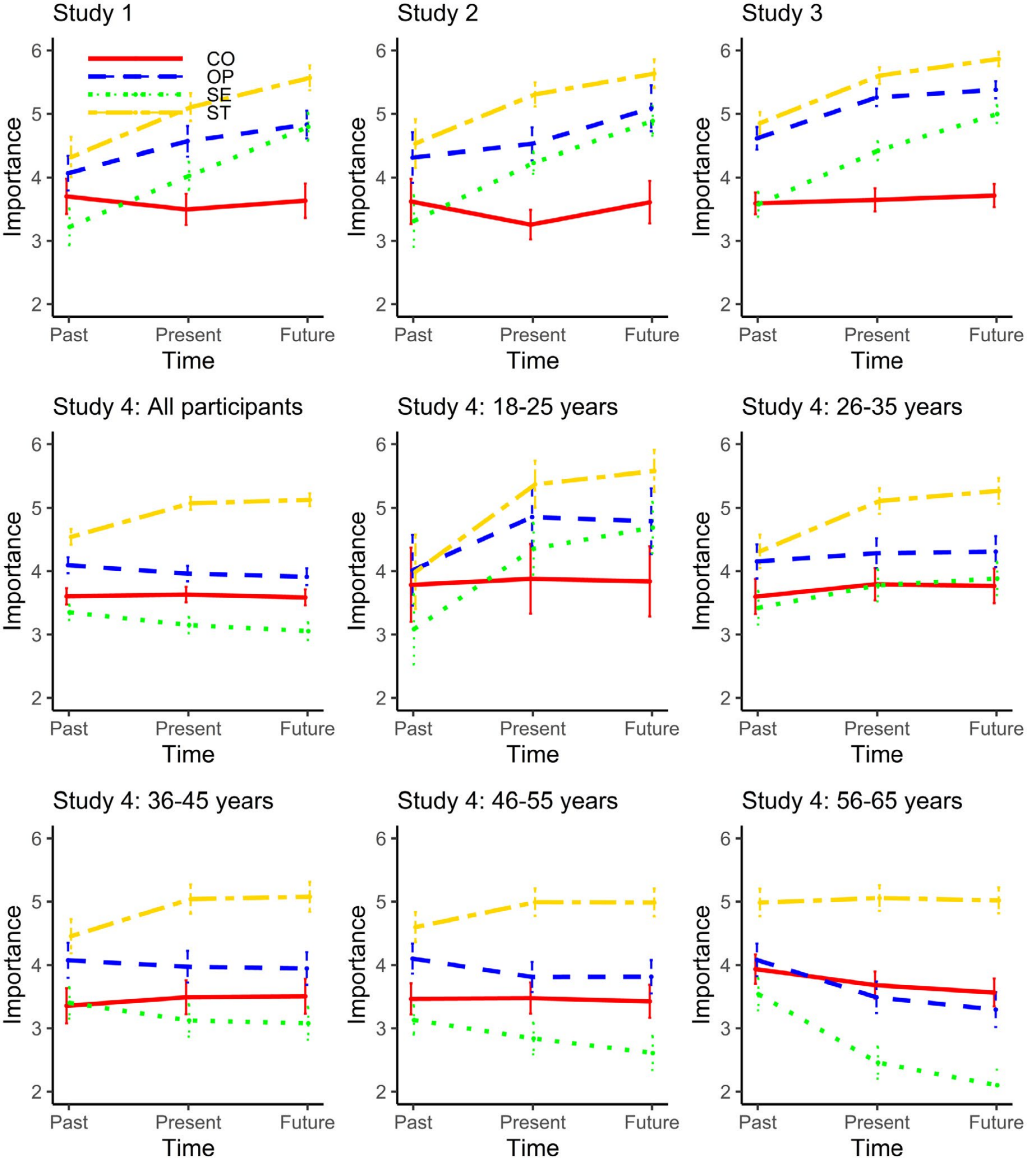
Absolute importance attributed to values over time (raw values); ST = Self‐transcendence, SE = Self‐enhancement, OP = Openness, CO = Conservation

To test for variability over time, a repeated‐measures analysis of variance (ANOVA) was conducted for each set of values. Figure 2 shows the raw data for each set of values over time for all four studies, with 95% confidence intervals to compare mean differences across time points. The within‐participants contrasts for self‐transcendence values showed a linear effect, *F*(1, 61) = 112.02, *p* < .001, *partial η* ^2^ = .65, and a weaker, but significant quadratic effect, *F*(1, 61) = 8.99, *p* < .01, *partial η* ^2^ = .13, such that the values were rated as increasing in importance over time, but to a lesser extent between current and future. The within‐participants contrasts for self‐enhancement values also showed a linear effect, *F*(1, 61) = 159.50, *p* < .001, *partial η* ^2^ = .72, but no significant quadratic effect, *F* < 1, *p* = .79, *partial η* ^2^ = .001; thus, these values were rated as increasing in importance over time. The within‐participants contrasts for openness values showed a linear effect, *F*(1, 60) = 22.32, *p* < .001, *partial η* ^2^ = .27, and no quadratic effect, *F*(1, 60) = 2.18, *p* = .15, *partial η* ^2^ = .03; thus, these values were rated as increasing in importance over time. The within‐participants contrasts for conservation values showed no significant linear effect, *F* < 1, *p* = .60, but there was a relatively weak quadratic effect, *F*(1, 60) = 4.70, *p* = .03, *partial η* ^2^ = .07, such that the values were rated as higher in both the past and the future, compared to current importance. We discuss the asymmetric effects between past, present, and future in the General Discussion.

### 2.4 Summary

These results indicate that participants perceived a shift over time in the importance they attribute to a range of values. There was a reliable tendency for most values to be seen as increasing in importance over time, with conservation values being the exception. Furthermore, participants did not show evidence for value oppositions over time. For instance, participants reported that both self‐transcendence and self‐enhancement values increase in importance to them over time. These two value types normally change in opposite directions: as either value type grows in importance, the other decreases in importance (Bardi et al., 2009; Maio, Pakizeh, et al., 2009). However, our participants reported these values as though they could pursue both sets of values concurrently. It is worth noting that this pattern does not simply reflect a process of general value inflation over time, because the conservation values did not show the same effect. These data thus provide intriguing evidence that participants are comfortable reporting the escalating pursuit of potentially conflicting values.

## 3 STUDY 2

Study 2 thus sought to address two methodological limitations of Study 1. First, having asked participants to respond to only opposing value types, it was now useful to ask participants to respond to all values, and hence, gather data that could be centered around each participant's mean value ratings, as Schwartz (2009) generally recommends for group mean comparisons. Second, it was important to test how responses to the temporal values measure compared to responses to a control condition. This was a novel comparison, with no specific a priori predictions about the direction of potential differences. However, this contrast would allow us to explore whether the temporal perspective offered by our new measure caused participants to change the level of importance participants attached to each value in the current context.

### 3.1 Method

#### 3.1.1 Participants

Participants were 92 undergraduate students at a British university (85 women, 7 men) who took part for course credit. They were between 18 and 26 years of age (*M* = 19).

#### 3.1.2 Design

A mixed design was used. Type of values measure was the between‐participant factor (temporal contrast, standard) and time (past, present, future) was the within‐participant factor.

#### 3.1.3 Procedure

Participants completed the study on computers in group sessions in a laboratory. They first completed the randomly assigned values measure. Next, for purposes unrelated to this report, participants completed items asking about behavioral examples related to the values.^3^ Finally, in the temporal contrast condition, participants were asked to note the ages they were contemplating when considering the past and future contexts^4^ and whether they imagined being employed, parenting, and in a relation in the future context. Afterward, participants were probed for suspicion, debriefed, and thanked for their time.

#### 3.1.4 Materials

The SVS scale was similar to Study 1, except that it contained 20 items representing both dimensions of the scale (self‐transcendence—self enhancement and openness—conservation), rather than just one of the dimensions (as in Study 1). Participants either completed the standard SVS, which asked only for their current rating of each value, or they completed the temporal contrast SVS, which asked for their rating of each value in the past, present, and future. As in Study 1, it was necessary to combine the five value scores representing each dimension at each time point. Reliability estimates at each time point were similar to Study 1: Cronbach's *α* varied from .66 to .78 for self‐transcendence values, from .51 to .78 for self‐enhancement values, from .74 to .81 for openness values, and from .62 to .72 for conservation values.

### 3.2 Results and discussion

Figure 2 presents the overall pattern of value ratings, excluding the control group. The figure reveals a similar pattern of results as obtained in Study 1. This conclusion was supported by repeated‐measures ANOVAs on the value ratings for each type of values. The within‐participants contrasts for self‐transcendence values revealed a linear effect, *F*(1, 45) = 60.72, *p* < .001, *partial η* ^2^ = .57, and a quadratic effect, *F*(1, 45) = 22.08, *p* < .001, *partial η* ^2^ = .33. The within‐participants contrasts for self‐enhancement values also showed a linear effect, *F*(1, 45) = 77.64, *p* < .001, *partial η* ^2^ = .63, and a weaker, but significant, quadratic effect, *F*(1, 45) = 4.44, *p* = .04, *partial η* ^2^ = .09. The within‐participants contrasts for openness values showed a linear effect, *F*(1, 45) = 27.40, *p* < .001, *partial η* ^2^ = .38, and a quadratic effect, *F*(1, 45) = 17.91, *p* < .001, *partial η* ^2^ = .29. The within‐participants contrasts for conservation values showed no significant linear effect, *F* < 1, *p* = .89, *partial η* ^2^ < .01, and a significant but relatively weak quadratic effect, *F*(1, 45) = 4.94, *p* = .03, *partial η* ^2^ = .10. Replicating the patterns from Study 1, the strongest effects over time were linear for self‐transcendence, self‐enhancement, and openness values, and a weak quadratic effect was detected for conservation values. Overall, the effects over time replicated and extended Study 1.

**FIGURE 3:**
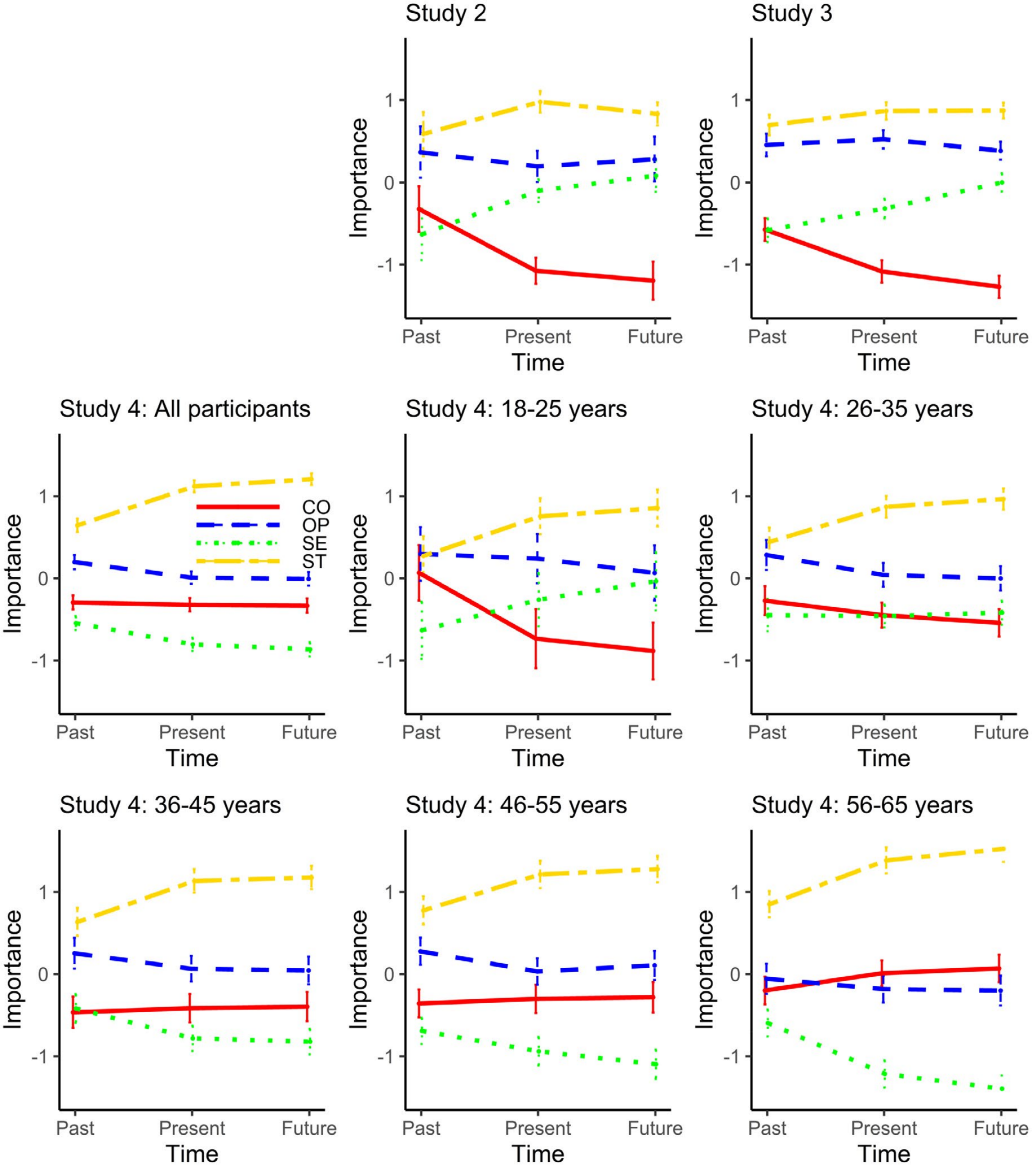
Relative importance attributed to values over time (centered value scores); ST = Self‐transcendence, SE = Self‐enhancement, OP = Openness, CO = Conservation. Note that the design of Study 1 prevented the calculation of centered scores

The second goal of the study was to use mean‐centered values scores. Figure 3 presents the pattern of centered data for each set of values for Studies 2, 3, and 4. For this study, repeated‐measures ANOVAs examined the mean‐centered data for each set of values. The within‐participants contrasts for self‐transcendence values revealed a significant linear effect, *F*(1, 45) = 4.85, *p* = .03, *partial η* ^2^ = .10, and a significant quadratic effect, *F*(1, 45) = 6.19, *p* = .02, *partial η* ^2^ = .12. The within‐participants contrasts for self‐enhancement values showed a linear effect, *F*(1, 45) = 24.81, *p* < .001, *partial η* ^2^ = .36, and no significant quadratic effect, *F* < 1, *p* = .74, *partial η* ^2^ < .01. The within‐participants contrasts for openness values showed no linear effect, *F* < 1, *p* = .52, *partial η* ^2^ = .01, and a significant quadratic effect, *F*(1, 45) = 5.92, *p* = .02, *partial η* ^2^ = .12. The within‐participants contrasts for conservation values showed a linear effect, *F*(1, 45) = 84.29, *p* < .001, *partial η* ^2^ = .65, and a quadratic effect, *F*(1, 45) = 27.81, *p* < .001, *partial η* ^2^ = .38.

These results provide an interesting contrast to the raw data. Looking at the relative (rather than absolute) value importance for each domain over time, the strongest effects are an upward effect for self‐enhancement values and a downward effect for conservation values. This pattern of data is particularly interesting given the wealth of evidence for the circular structure of the values contained within the SVS. The raw data from Studies 1 and 2 suggest that participants did not consciously see the oppositional nature of each set of values. The mean‐centered data in Study 2 also suggest that such opposition is not present, even though centering focuses on relative changes in value importance. Thus, even though centering affects the plotted angle of value change, more crucial is that the patterns between values indicate again that perceptions of value change over time do not reveal the same motivational relations between values as have been found when examining actual value changes over time (Bardi et al., 2009; Vecchione et al., 2016).^5^

The second goal of this study was to test whether completion of the temporal contrast ratings of values yields current ratings that differ from current ratings that are made without the temporal contrast being salient. As Table 1 illustrates, participants in the temporal contrast condition raised the importance of their values in the present over participants in the control condition, although this effect was reliable only for openness values. That is, thinking about values over time enhanced the perceived importance of openness values in the current context.

**TABLE 1:** Comparing data from the temporal contrast SVS to standard SVS

	Temporal contrast	Standard	*t*	Cohen's *d*
	*M* (*SE*)	*M* (*SE*)		
Self‐transcendence	5.37 (0.14)	5.24 (0.13)	−0.70	−0.15
Self‐enhancement	4.28 (0.14)	4.18 (0.10)	−0.60	−0.13
Openness	5.02 (0.17)	4.03 (0.17)	−4.10**	−0.86
Conservation	3.47 (0.17)	3.04 (0.16)	−1.87^†^	−0.39
All Values	4.54 (0.11)	4.12 (0.09)	−2.88**	−0.61
Self‐transcendence (centered)	0.84 (0.08)	1.12 (0.10)	2.15*	0.45
Self‐enhancement (centered)	−0.25 (0.10)	0.05 (0.09)	2.25*	0.47
Openness (centered)	0.48 (0.12)	−0.09 (0.14)	−3.10**	−0.65
Conservation (centered)	−1.07 (0.12)	−1.08 (0.11)	−0.11	−0.02

Note

Higher means represent greater importance.

^†^ *p* < .10;

* *p* < .05;

** *p* < .01.

We checked whether these differences from the control condition also arose in the mean‐centered value scores. Table 1 shows that the centered data revealed different effects from the raw value scores. Participants who considered their values over time significantly increased the importance they attached to openness values, similar to the raw data. However, as the centered data within a time point are necessarily relative in nature, decreases in centered value importance are needed to balance any such change. Here, the decreases in centered importance occurred for self‐transcendence and self‐enhancement values, but this was not accompanied by a decrease in the importance they attached to the motivationally opposing, conservation values. Interestingly, these data also show participants did not report conflict across the value dimensions. In comparison to the control group, participants who thought about the temporal trajectories of their values clearly increased the importance they attached to openness values in the current context, but manifested decreases only on the independent value dimension from self‐transcendence to self‐enhancement and not on the opposing conservation values.

### 3.3 Summary

Study 2 replicated the patterns found in Study 1. Values were again perceived as increasing over time, except when we examined conservation values. The mean‐centered data revealed a polarized version of the pattern, with participants projecting a rise in self‐enhancement values and a fall in conservation values over time. Of importance, neither the raw data nor the centered data provided evidence that the participants saw theoretically opposing values as being in competition in their changes over time. Finally, completion of the temporal contrast significantly enhanced the perceived importance of openness values in the current context, compared to when participants completed the standard values measure.

## 4 STUDY 3

The first two studies provided consistent evidence that participants saw the majority of their values increasing in importance over time, without counterbalancing upward changes in some values with downward changes in the motivationally opposing dimension. Study 3 attempted to replicate this pattern in a larger sample, while testing whether the stability of participants’ values over time is associated with subjective well‐being, and whether this association differs across value types. This analysis is novel and exploratory, but we hypothesized that participants who report more variability in their values over time may also report lower well‐being, as such variability could reflect less satisfaction with chosen value importance across the lifespan. Variability was operationalized with the within‐person standard deviation of past, present, and future values, averaged separately for each of the four higher‐order value types.

### 4.1 Method

#### 4.1.1 Participants

Participants were 198 first‐year undergraduate students at a British university (175 women, 23 men) who took part in a pretest research session during their induction week. They were between 17 and 50 years of age (*M* = 19).

#### 4.1.2 Design

A correlational design was used. All the participants completed the temporal contrast measure of values and three measures of well‐being.

#### 4.1.3 Procedure

The research was presented as two separate studies within a large testing session, wherein participants completed a diverse range of psychological measures on computer for other researchers, in addition to the measures used in our research. Accordingly, participants were free to complete the measures in any order they preferred. Participants were debriefed and thanked after completion of the session.

#### 4.1.4 Materials

The same temporal contrast measure of values was used as in Study 2. In addition, three scales were used to measure well‐being. The first measure was the Satisfaction with Life Scale (SWLS), which measures cognitive judgments of well‐being (Diener et al., 1985). Example items included “In most ways, my life is close to ideal” and “So far, I have gotten the important things I want in life.” Participants responded using a scale from 1 (*strongly disagree*) to 7 (*strongly agree*), and the five items formed a single reliable factor (*α* = .88). The second measure was the Scale of Positive and Negative Experience (SPANE), which identifies current emotional well‐being (Diener et al., 2010). Example items for positive emotions included “happy,” “joyful,” and “good” and example items for negative emotions included “sad,” “angry,” and “bad.” Participants were asked to think about how much they had experienced each feeling over the last four weeks. Participants responded using a scale from 1 (*very rarely or never*) to 5 (*very often or always*). The positive emotions (*α* = .88) and negative emotions (*α* = .84) formed reliable factors, as did the combination of both scales (*α* = .87) (to calculate the SPANE overall score, the negative items are reverse coded). The third measure was the Flourishing Scale (FS), which captures the extent to which people feel they are functioning well in important areas of human life (Diener et al., 2010). Example items included “I lead a purposeful and meaningful life” and “I am competent and capable in the activities that are important to me.” Participants responded using a scale from 1 (*strongly disagree*) to 7 (*strongly agree*), and the five items formed a single reliable factor (*α* = .86). We also calculated a well‐being index, which is a composite of the SWLS, SPANE, and FS after each item has been standardized (*α* = .84).

### 4.2 Results and discussion

We tested whether the raw value scores replicated the effects from the first two studies. The pattern of raw data neatly aligned with the previous results (see Figure 2). A repeated‐measures ANOVA for self‐transcendence values showed a linear effect, *F* (1, 197) = 202.30, *p* < .001, *partial η* ^2^ = .51, and a weaker quadratic effect, *F* (1, 197) = 49.32, *p* < .001, *partial η* ^2^ = .20. The within‐participants contrasts for self‐enhancement values also showed a linear effect, *F* (1, 197) = 265.41, *p* < .001, *partial η* ^2^ = .57, and a weaker, but reliable, quadratic effect, *F* (1, 197) = 9.63, *p* < .01, *partial η* ^2^ = .05. The within‐participants contrasts for openness values showed a linear effect, *F* (1, 197) = 104.61, *p* < .001, *partial η* ^2^ = .35, and a quadratic effect, *F* (1, 197) = 43.12, *p* < .001, *partial η* ^2^ = .18. The within‐participants contrasts for conservation values showed a marginally significant linear effect, *F* (1, 197) = 3.47, *p* = .06, *partial η* ^2^ = .02, and no significant quadratic effect, *F* < 1, *p* = .86, *partial η* ^2^ < .01.

Having found consistency in the raw value scores across studies, we tested whether the pattern of centred data from Study 2 was also replicated (see Figure 3). A repeated‐measures analysis of variance (ANOVA) on the self‐transcendence values revealed significant linear, *F* (1, 197) = 13.17, *p* < .001, *partial η* ^2^ = .06, and quadratic effects, *F* (1, 197) = 9.70, *p* < .01, *partial η* ^2^ = .05. The within‐participants contrasts for self‐enhancement values showed a linear effect, *F* (1, 197) = 79.00, *p* < .001, *partial η* ^2^ = .29, and no significant quadratic effect, *F* < 1, *p* = .50, *partial η* ^2^ < .01. The within‐participants contrasts for openness values showed no linear effect, *F* < 1, *p* = .25, *partial η* ^2^ = .01 and a significant quadratic effect, *F* (1, 197) = 10.67, *p* < .01, *partial η* ^2^ = .05. The within‐participants contrasts for conservation values showed a linear, *F* (1, 197) = 156.74, *p* < .001, *partial η* ^2^ = .44, and a quadratic effect, *F* (1, 197) = 37.20, *p* < .001, *partial η* ^2^ = .16.

These raw and mean centered effects were very similar to those in Study 2, providing a robust foundation for our second aim, which was to look at the relation between perceived value variability and well‐being (see Tables S1 and S2 for zero‐order correlations at each time point). For the raw value scores, while in the hypothesized direction, associations between values and the well‐being measures were inconsistent and mostly nonsignificant (see Table S9). The only exception was a marginal correlation between conservation values and satisfaction with life, *r*(196) = −.14, *p* = .059. Similarly, for the centered value scores, most results were in the hypothesized direction but nonsignificant (see Table S9). However, variability in openness was associated with satisfaction with life, *r*(198) = −.19, *p* = .007; and variability in self‐transcendence was marginally associated with satisfaction with life, *r*(198) = −.12, *p* = .085.

## 5 SUMMARY

The results from Study 3 replicated the patterns obtained in the first two studies. These patterns continue to show that people do not encompass motivational oppositions between values within subjective perceptions of their own value change over time. In addition, we found that perceived variability in openness values was associated with lower well‐being—a finding we test again in Study 4 and address further in the General Discussion.

## 6 STUDY 4

Study 4 aimed to replicate and extend Studies 1–3 in a larger sample from the general public. The aims were fourfold. First, we explored whether the results would be different if participants think about 10 years in the past and future, rather than at a self‐chosen time‐point. Consequently, we compared responses to the past, present, and future between a condition that asked participants to think about 10 years in the past and in the future versus a condition wherein the past and future time points were self‐chosen (as in Studies 1–3). Second, we explored whether participant age is associated with how participants perceive their past and future values, because goals and perspective change later in our lifespan (Carstensen, Isaacowitz, & Charles, 1999), potentially altering our predictions for future value change. Third, we tested whether past and future values would predict past and future self‐reported behavior better than present values. If future values explain variance above and beyond present values, this would make a case for measuring future values when the research aim is to predict expected behavior at the future time point. This possibility would be interesting in light of evidence that values are stronger predictors of distant future behavior intentions than temporally proximal behavioral intentions (Eyal et al., 2009). Fourth, we tested whether value variability would predict well‐being in a more diverse sample than in Study 3.

### 6.1 Method

#### 6.1.1 Participants

To explore age effects across the lifespan, we aimed to recruit 100 participants from each of the following five age groups: 18–25 years, 26–35 years, 36–45 year, 46–55 years, and 56–65 years. However, because we explicitly excluded students (to test for generalizability beyond a student sample), our recruitment agency was only able to recruit 40 participants from the youngest age group. We recruited 120 participants from each of the remaining four age groups, after all participants who failed an instructional manipulation check item were excluded. As an additional quality check, we tested whether internal consistencies (i.e., alphas) for several of the response scales would be lower for the fastest or slowest 5% of participants. As this was not the case, we collapsed across response duration. Participants were 520 people from the general public (314 women, 200 men, 3 other, 3 prefer not to say) who took part online (*M_age_* = 44). One‐hundred fifty‐six participants had an educational degree below an Advanced level U.K. secondary school leaving qualification (A level), 121 were holding an A‐level as their highest qualification, and 243 were holding a university degree (bachelor, master, PhD).

#### 6.1.2 Design

A mixed design was used, with temporal constraints (yes, no) and age group as the between‐participant factors and time (past, present, future) as the within‐participant factor.

#### 6.1.3 Procedure

Participants were contacted through a paid recruitment service. Participants first completed the temporal contrast measure of values, then well‐being measures and a behavior measure, followed by items assessing political perspective^6^ and demographics. Upon completion, participants were debriefed and thanked for their time.

#### 6.1.4 Materials

In the no‐constraint condition, we used the same temporal contrast measure of values as in Study 3. In the time‐constraint condition, participants were instructed to think about a time point 10 years ago, the present, and in 10 years' time. To measure well‐being, we used the same three scales as in Study 3: Satisfaction with Life Scale (Diener et al., 1985), Scale of Positive and Negative Experience (Diener et al., 2010), and Flourishing Scale (Diener et al., 2010). In addition, we measured optimism with the revised 6‐item Life Orientation Scale (Scheier et al., 1994). Example items included “In uncertain times, I usually expect the best" and “If something can go wrong for me, it will” (reversed coded). Participants responded using a scale from 1 (*strongly disagree*) to 5 (*strongly agree*). To reduce blind responding, four filler items were included in this scale.

To assess past, present, and future behavior, we derived four behaviors for each of the four higher order values that would be relevant to the U.K. public, similar to Bardi and Schwartz (2003). Example behaviors include “Supporting charities with your time or money” (self‐transcendence), “taking interest in British history” (conservation), “making decisions on behalf of other people” (self‐enhancement), and “buying new foods that you haven't tried before” (openness). All 16 behaviors can be found in Appendix B. Internal consistencies of all scales are reported in Tables S7 and S8.

### 6.2 Results and discussion

First, we tested whether temporal constraints affected the past, present, and future values, using a series of between‐subject *t* tests. None of the *t* tests were significant for either raw or centered data (Tables S3 and S4), thus, for all remaining analyses we collapsed across both conditions. Also, no gender differences were found, so we collapsed across gender.

This study's second aim was to test for age effects. To do this, we performed mixed‐ANOVAs on each of the four higher‐order values, with age group (18–25, 26–35, 36–45, 46–55, and 56–65 years) as the between‐participant factor and time (past, present, future) as the within‐participant factor.^7^ The interactions in all four mixed‐ANOVAs were significant, *p* < .001 (see Supporting Information for details). We therefore tested in a series of within‐subject ANOVAs whether the pattern found in Studies 1–3 replicated across age groups, first using the raw scores (Figure 2 and Table S5). To simultaneously avoid a focus on small contrasts and to control for multiple‐comparisons, we changed our alpha‐level from .05 to .005.

For self‐transcendence values, we found significant linear and quadratic contrasts for all but the oldest age group: participants rated their future self‐transcendence values as most important and their past values as least important. For openness values, we found mixed results. We found linear effects for participants between 18–25 years and 56–65 years, but in a reversed order: while the youngest participants rated their present openness values higher than their past values, this pattern was the opposite for the oldest of our participants. Also, for participants between 18 and 25‐years, we found a quadratic effect: present openness values were rated as most important. For self‐enhancement values, we found linear contrasts for all age groups except for the 36–45‐year‐old participants. Interestingly, while 18–25 and 26–35‐year‐old participants rated their past self‐enhancement values as least important and their future self‐enhancement values as most important, this pattern was reversed for the three older age groups. Additionally, we found significant quadratic contrasts for the 18–25‐year‐old participants and the 56–65‐year‐old participants, who rated their present and future self‐enhancement values to be more similar on average than their past and present values. For conservation values, we only found significant linear contrasts for the oldest group: they believed their conservation values would decline in importance.

Next, we tested whether we find the same effects using the centered scores (Figure 3 and Table S6), again using a stricter alpha‐level of .005. For self‐transcendence values, we found significant linear and quadratic contrasts for all groups: participants rated their future self‐transcendence values as most, and their past values as least, important. For openness values, we again found mixed results. While all age groups rated their past openness values as higher in importance than their future values, with the present values mainly in between, this trend only reached statistical significance for participants between 26 and 35‐years. For participants between 46 and 55‐years, we found a quadratic but no linear effect: present openness values were rated as least important. For self‐enhancement values, we found linear contrasts for all age groups except for the 26–35‐year‐olds. Interestingly, while 18–25‐year‐old participants rated their past self‐enhancement values as least important and their future self‐enhancement values as most important (with the present values in the middle), this pattern was reversed for the three older age groups. Additionally, we found significant quadratic contrasts for the 36–45‐year‐old participants and the 56–65‐year‐old participants, who rated their present and future self‐enhancement values to be more similar on average than the past and present values. For conservation values, we found linear contrasts for the two youngest age groups and the oldest group. While the two youngest age groups believed that their conservation values would become less important over time, the oldest age group believed their conservation values would become more important. For the two youngest groups, we also found significant quadratic trends, with the present and future values again being perceived as more similar than the past and present values.

Overall, we again replicated the prior evidence from Studies 1–3 for the youngest age group in both the raw value ratings and the centered value ratings, but this pattern differed for older participants. Given this context, we set out to meet the study's third aim by correlating past, present, and future values with past, present, and future self‐reported behaviors. We report our findings across all age groups because age did not moderate the association between values and self‐reported behavior, neither for raw value and raw behavior scores, nor centered values and centered behavior scores, (*p*s ≥ .05). The correlations between values and self‐reported behavior were in general large for the raw scores and somewhat smaller for the centered scores (Tables S7 and S8). Overall, the correlations were strongest if value and time were consistent (e.g., past values with past self‐reported behavior of the same value).

To explore whether future raw values predict future raw behavioral intentions better than present values, we ran four regression analyses—one per higher order value—with future intentions as the outcome and the present and future values as predictors. Future self‐transcendence‐related intentions were neither predicted by present nor future self‐transcendence values (*β*_p_ = .17, *p* = .13, and *β*_f_ = .17, *p* = .14; subscript “p” refers to present and “f” to future values). In contrast, future openness intentions were not predicted by present values, but were predicted by future openness values (*β*_p_ = −.08, *p* = .35, and *β*_f_ = .38, *p* < .001). Future self‐enhancement intentions were predicted strongly by future self‐enhancement values, and somewhat by present values (*β*_p_ = −.25, *p* = .004, and *β*_f_ = .68, *p* < .001). Finally, future conservation intentions were only predicted by present, but not future values (*β*_p_ = .52, *p* < .001, and *β*_f_ = −.22, *p* = .07). Overall, future values are uniquely relevant in predicting future intended openness and self‐enhancement‐related intentions, while present values were uniquely linked to future intentions for conservation behavior.

We ran the same regression analyses for the centered value and self‐reported behavior scores. Future self‐transcendence‐related intentions were predicted by future but not by present self‐transcendence values (*β*_p_ = .08, *p* = .30, and *β*_f_ = .19, *p* = .02). Similarly, future openness‐related intentions were predicted by future but not present openness values (*β*_p_ = −.03, *p* = .64, and *β*_f_ = .28, *p* < .001). Future self‐enhancement‐related intentions were predicted strongly by future self‐enhancement values and more weakly by present self‐enhancement values (*β*_p_ = −.22, *p* < .001, and *β*_f_ = .59, *p* < .001). Finally, future conservation‐related intentions were predicted both by future and present conservation values (*β*_p_ = .17, *p* = .04, and *β*_f_ = .21, *p* = .007). In sum, centered future values explained significant variance in future intentions above present values across all four higher order value types.

The study's fourth aim was to test whether a link between value variability and well‐being could be established in our more diverse sample (compared to Study 3) (see Tables S7 and S8 for zero‐order correlations at each time point). Variability was again operationalized with the standard deviation of past, present, and future values, separately for each of the four higher‐order value types. For the raw value scores, variability in openness was consistently associated with less satisfaction with life, *r*(517) = −.20, *p* < .001, less positive emotions, *r*(517) = −.13, *p* = .004, more negative emotions, *r*(517) = .11, *p* = .01, less flourishing, *r*(517) = −.14, *p* = .001, and less optimism, *r*(517) = −.11, *p* = .01. This suggests that people whose openness values are more variable report lower well‐being. Associations between the other three values and the well‐being measures were inconsistent and mostly nonsignificant, *r*s ≤ .09, *p*s ≥ .03. For the centered value scores, variability in openness was again consistently associated with satisfaction with life, *r*(517) = −.15, *p* < .001, positive emotions, *r*(517) = −.10, *p* = .03, negative emotions, *r*(517) = .13, *p* = .002, and flourishing, *r*(517) = −.09, *p* = .04. Variability in self‐transcendence and conservation values were also negatively associated with satisfaction with life, *r*(518) = −.12, *p* = .004, and *r*(517) = −.10, *p* = .02. In sum, these results demonstrate an important relationship between perceived temporal value variability and well‐being, with replication of the association between variability in openness values and satisfaction with life found in Study 3.

### 6.3 Summary

The results from Study 4 mainly replicated the temporal value patterns obtained in the first three studies. Instructing participants to think about their values at a specific time point did not change the pattern of value ratings across past, present, and future. Interestingly, however, the pattern of results was moderated by age. Younger participants (nonstudents; under 35) assumed that their self‐enhancement values would increase, while older participants assumed they would decrease; the reversed pattern was found for conservation values. Furthermore, future values proved useful in predicting future behavioral intentions, even when controlling for present values. Finally, participants with greater variability in values, particularly in the openness dimension, reported lower well‐being.

## 7 GENERAL DISCUSSION

In relation to our primary aims, the four studies provided consistent evidence that people perceive their values as changing over time. At the same time, perceived value trajectories rarely express the motivational conflict seen across diverse research paradigms. Of particular relevance, a number of studies have indicated that self‐transcendence and self‐enhancement values change in opposite directions, as do openness and conservation values (Bardi et al., 2009; Maio, Pakizeh, et al., 2009). In all four of the present studies, however, younger participants viewed their self‐transcendence, self‐enhancement, and openness values as *all* becoming more important over time. Only the conservation values were perceived as unchanging. The absence of motivational conflict in value trajectories occurred when we examined both raw and centered data. Even when we isolated directly opposing values (Study 1), participants still did not evidence the potential opposition over time. Together, these findings indicate that the motivational conflicts that are expressed in values are not reflected in people's subjective perceptions of how their values change over time. When considering values over time, younger people (under 35) expect that most values can simultaneously grow in importance.

Nonetheless, it was not the case that asking about values from a temporal perspective merely invoked an upward lift in all values, nor for all ages. Across the lifespan, conservation values were relatively stable in importance. Unlike the other values, conservation values focus on protecting the status quo by protecting safety and security. In contrast, self‐transcendence, self‐enhancement, and openness values have the capacity to engage growth motivations (Schwartz et al., 2012). Thus, the value trajectories reflect a need for progress and growth and not a need for further protection of the status quo.

A final important point about the value trajectories is that the differences between past and present were generally larger than between present and future. This result replicates the aforementioned research by Quoidbach and colleagues (2013), showing variability in values decreases with age. While our methodology asked the participants to draw trajectories across three time points, and we thus cannot isolate the time points independently, future work could examine the past‐present and present‐future differences in between‐participant designs, to confirm how these gaps vary when projections are made in only one temporal direction (toward past or future).

In terms of our secondary aims, our findings indicate that the temporal values measure matters in at least four further ways. First, assessing values over time led to increased importance being attached to openness values—it thus offers a novel intervention for anyone interested in making openness values salient. Second, we found raised correlations between variability in openness values and well‐being in Studies 3 and 4, demonstrating that greater variance in values over time is associated with lower current satisfaction with life. Third, we found in Study 4 that the prediction of future intentions is enhanced by asking about future values. This evidence extends prior observations that it is important to consider temporal focus in questions predicting behavioral intentions from values (Eyal et al., 2009). Here we show that the temporal match can be augmented by placing the values items in the same temporal frame as the behavioral intentions items. Fourth, we found that the subjective value trajectories vary between people of different ages. This result fits prior theory on changes in socioemotional selectivity as we age (Carstensen et al., 1999).

### 7.1 Limitations and future directions

Although our research establishes a consistent pattern of value trajectories and demonstrates some important consequences of these trajectories, this novel method of assessing values over time is necessarily at an early, exploratory, and somewhat descriptive stage. There are thus a number of limitations to consider. While we have shown how values are perceived as changing over time and across age groups, we cannot yet state precisely why this happens. Future research should address some important theoretical and empirical questions that follow from our findings.

First, it would be valuable to consider what contexts people think about when judging their values over time. We did not find any difference by constraining the past and future time points (Study 4), which implies our initially situationally unconstrained temporal methodology did not lead to greater use of concrete instantiations compared to standard measures. But this possibility cannot be ruled out completely, and future studies could ask people to report the cognitions underlying their perceived value trajectories.

The concern of participants using varying contexts to decide upon value importance would of course also be relevant to the standard measures, where tensions are regularly found (Schwartz et al., 2012). However, contextual shifts could be valuable in understanding why younger people reported upward change in theoretically opposing values—does thinking about values in the future allow for greater variability in the range of instantiation contexts considered (e.g., I will be more concerned for others in a family context, but I will also be more concerned with myself in a vocational context)? It would be valuable to know whether considering values over time leads to instantiations that are more concrete or diverse, and whether any such tendencies are also moderated by age. Additionally, our designs did not directly ask participants about whether or not they perceived opposition in pursuing conflicting values (e.g., wealth and equality). To increase our understanding of how people view such conflicts, it would therefore be useful to examine the extent to which participants see this as problematic when directly confronted.

Second, to allow comparable analyses across our samples we used behavioral intentions via top‐down constructed items to assess the predictive validity of future value importance. While our results demonstrate that future values show promise as a better predictor of intentions than current values, these findings would benefit from extension. Similar to the previous point about value instantiations, further research could explore which contexts participants are considering when thinking about future actions. All values measures assess assigned importance to broad and abstract ideals, however, past research has shown that *the effects* of values also depend a great deal on the specific concrete instantiations of the values that are brought to mind (Maio, 2016; Maio, Hahn, et al., 2009). A further useful test would be to examine whether future values predict behavior better than present values in a longitudinal design.

Third, the changing trajectories across age groups show where life stage has an impact on the perception of values over time. Values can change for two interconnected reasons: internal (a shift in motivation) and external (a shift in context). The suggested future directions above could also be used to explore how participants approach the temporal values measure across the lifespan. Our finding that older participants, unlike their younger counterparts, do report opposing trajectories for self‐enhancement and self‐transcendence is worthy of particular attention. Does greater life experience lead to people realizing they cannot “have it all” (a shift in internal motivation)? Or do changes in life circumstances lead to different things mattering (a shift in external context)? Given the importance of understanding the social context within which values and well‐being interact (Burr et al., 2011), it would be informative to track how perceived variability in values over time might be moderated by different life experiences.

Fourth, our findings relating value trajectories to well‐being are intriguing, though the effect sizes are small, in line with previous research (e.g., Oishi et al., 1999). The fact that the relationships appear more consistent in the larger, more representative sample is reassuring in terms of reliability. Our operationalization is, however, a general and conservative test of this association, since it cannot account for how participants interpret the changes over time. A more detailed examination could ask participants to assess whether they see their changes as generally positive (e.g., fulfilled desire, aspiration) or negative (e.g., regret, dissatisfaction), and any such moderation would improve our understanding of how variability in values can impact upon well‐being. It is also noteworthy that, in both studies examining well‐being, we replicated research showing higher self‐transcendence and openness values in general correlate with higher levels of well‐being (Burr et al., 2011; Deci & Ryan, 1995; Kasser & Ahuvia, 2002; Sortheix & Schwartz, 2017).

There are further important implications for understanding temporal value projections alongside other research domains. For instance, people can show dispositional variation in their tendency to focus on different temporal parts of their lives (Barber et al., 2009), and it would be worth seeing if such tendencies moderate our findings by using a standardized measure of time perspective (Zimbardo & Boyd, 1999). For example, those who tend to focus more on the future might report smaller discrepancies between current and future value importance, or find it easier to accommodate potential value conflict in their future values. Indeed, the perceived distance between the current self and other time points matters in self‐projection (Wilson et al., 2012), and there may be implications for well‐being, as our findings linking value variability and well‐being indicate.

Furthermore, many studies have shown that temporal perspective can be manipulated for potential positive impact. Creativity, self‐control, and life‐satisfaction have all been shown to increase as a result of taking a more abstract, distant perspective (Burgoon et al., 2013). Similarly, various methods of focussing on the future have increased long‐term thinking (Liu & Aaker, 2007), academic achievement (Barber et al., 2009), idealism (Kivetz & Tyler, 2007), pro‐environmental engagement (Pahl & Bauer, 2013), and self‐transcendence values (Joireman & Duell, 2005), while also protecting against negative affective responses (Namkoong & Henderson, 2014). Our data demonstrate a strong increase in the importance attached to openness values when using the temporal values measure (Study 2), which is a promising indicator of the potential to use this method to encourage horizon scanning.

### 7.2 Conclusion

In sum, the four studies reported here provide consistent evidence that people see their values as changing over time. With the exception of conservation values, younger people generally believe that they are increasing the importance of all their values over time. Most individuals' visions of change are not consistent with the tensions across value dimensions that have been consistently found in previous research. This inconsistency means younger people might be unaware of the potential problems of trying to concurrently pursue opposing life goals. However, people in older age groups report a different pattern that importantly shows some evidence of recognizing value opposition, with rising self‐transcendence values being somewhat offset by falling self‐enhancement values. These data open a number of intriguing empirical pathways questioning our perception of value change over time and across the lifespan, while showing that a temporal measure of values can be useful in predicting well‐being and future intentions, as well as encouraging openness. With further development and research, the method of asking participants to reflect on their values in the past, now, and in the future may allow people to use their values more powerfully and in a more fulfilling manner.

## CONFLICT OF INTEREST

The authors declared no potential conflicting interests with respect to the research, authorship and/or publication of this article.

## ACKNOWLEDGMENTS

Preparation of this manuscript was supported by the Economic and Social Research Council UK (ESRC‐1013799). The authors would also like to thank Geoff Haddock for his support in the production of this paper.

## Supporting information

Supplementary Material

Supplementary Material

Supplementary Material

## Data Availability

The data that support the findings of this study are openly available in OSF at: https://osf.io/j56tr/?view_only=29857ace11e14caf92400d0d31f7adb4.

## ENDNOTES

^1^
Analyses for Study 1and Study 2 revealed the PFC had no significant effects, it is thus mentioned here for procedural completeness, but is not discussed in the results sections.

^2^
We also piloted a scrambled sentences priming manipulation in Study 1, mentioned here for procedural completeness. Analyses revealed no significant effects. No conclusions can be drawn from these data, as the study was not adequately powered to detect the effect sizes that are generally reported in the values priming literature. It is thus not discussed in the results section.

^3^
Ten items provided scenarios that reflected germane behaviors for a student sample (e.g., deciding which career to pursue; voting in a student election). These items asked participants to think about each scenario and consider how their decision would be driven by personal interest versus the needs of others. These items were designed to examine behavioral examples in the self‐transcendence—self‐enhancement dimension of values, *after* our critical values‐over‐time measure had been completed. Answers were provided on a scale from 1 (*entirely driven by personal interests*) to 9 (*entirely driven by the needs of others)*. They are mentioned here for procedural completeness, but are not discussed in the results section.

^4^
Analyses were not affected by the ages participants provided and are thus not discussed further. Additional analyses describing relevant zero‐order correlations are available in Supporting Information.

^5^
We also assessed whether the typical circular value structure holds within each time point of the temporal values measure. Our multidimensional scaling analyses suggest that it does (see Figures S1–S4).

^6^
The political measure was of secondary interest and not discussed further here for parsimony. Results are in Supporting Information (Tables S7 and S8).

^7^
We report a series of ANOVAs rather than moderated regressions with age as a continuous predictor to be consistent with Studies 1–3 and to allow past, present, and future time points to be evaluated simultaneously, as the task required. However, for those interested in analyses that use age as a continuous variable, moderated regressions with age as the predictor, time as a moderator (past vs. present, and present vs. future), and value importance as outcome, these are reported in the Supporting Information (Tables S10 and S11 for raw and centred value scores, respectively).
