# Supplementary material for: Perceptions of values over time and why they matter
Source: J Pers. 2020 Dec 5;89(4):689–705. doi: 10.1111/jopy.12608 (PMC8518993; doi:10.1111/jopy.12608)
Supplement: Supplementary file 1 — Supplementary Material [file JOPY-89-689-s001.docx]

**Appendix A**

Example of temporal values measure (Study 1)

**Task 2**

**Values list**

You will now be presented with a list of ten values. People tend to vary in their ratings of the relative importance of these values. We would like you to rate each value in three ways:

1. How important you think this value has been as a guiding principle in your life to you in the past.
2. How important you think this value is as a guiding principle in your life now.
3. How important you think this value will be as a guiding principle in your life in the future.

| **Wealth** (making money) | Opposed to my values | Not important |  |  | Moderately important |  |  | Very important | Of supreme importance |
| --- | --- | --- | --- | --- | --- | --- | --- | --- | --- |
|  | -1 | 0 | 1 | 2 | 3 | 4 | 5 | 6 | 7 |
| In the past | 🞏 | 🞏 | 🞏 | 🞏 | 🞏 | 🞏 | 🞏 | 🞏 | 🞏 |
| Now | 🞏 | 🞏 | 🞏 | 🞏 | 🞏 | 🞏 | 🞏 | 🞏 | 🞏 |
| In the future | 🞏 | 🞏 | 🞏 | 🞏 | 🞏 | 🞏 | 🞏 | 🞏 | 🞏 |
|  |  |  |  |  |  |  |  |  |  |
| **Power** (control over others, dominance) | Opposed to my values | Not important |  |  | Moderately important |  |  | Very important | Of supreme importance |
|  | -1 | 0 | 1 | 2 | 3 | 4 | 5 | 6 | 7 |
| In the past | 🞏 | 🞏 | 🞏 | 🞏 | 🞏 | 🞏 | 🞏 | 🞏 | 🞏 |
| Now | 🞏 | 🞏 | 🞏 | 🞏 | 🞏 | 🞏 | 🞏 | 🞏 | 🞏 |
| In the future | 🞏 | 🞏 | 🞏 | 🞏 | 🞏 | 🞏 | 🞏 | 🞏 | 🞏 |
|  |  |  |  |  |  |  |  |  |  |
| **Ambition** (hardworking, aspiring) | Opposed to my values | Not important |  |  | Moderately important |  |  | Very important | Of supreme importance |
|  | -1 | 0 | 1 | 2 | 3 | 4 | 5 | 6 | 7 |
| In the past | 🞏 | 🞏 | 🞏 | 🞏 | 🞏 | 🞏 | 🞏 | 🞏 | 🞏 |
| Now | 🞏 | 🞏 | 🞏 | 🞏 | 🞏 | 🞏 | 🞏 | 🞏 | 🞏 |
| In the future | 🞏 | 🞏 | 🞏 | 🞏 | 🞏 | 🞏 | 🞏 | 🞏 | 🞏 |
|  |  |  |  |  |  |  |  |  |  |
| **Success** (achieving goals) | Opposed to my values | Not important |  |  | Moderately important |  |  | Very important | Of supreme importance |
|  | -1 | 0 | 1 | 2 | 3 | 4 | 5 | 6 | 7 |
| In the past | 🞏 | 🞏 | 🞏 | 🞏 | 🞏 | 🞏 | 🞏 | 🞏 | 🞏 |
| Now | 🞏 | 🞏 | 🞏 | 🞏 | 🞏 | 🞏 | 🞏 | 🞏 | 🞏 |
| In the future | 🞏 | 🞏 | 🞏 | 🞏 | 🞏 | 🞏 | 🞏 | 🞏 | 🞏 |
|  |  |  |  |  |  |  |  |  |  |
| **Influence** (having an impact on people and events) | Opposed to my values | Not important |  |  | Moderately important |  |  | Very important | Of supreme importance |
|  | -1 | 0 | 1 | 2 | 3 | 4 | 5 | 6 | 7 |
| In the past | 🞏 | 🞏 | 🞏 | 🞏 | 🞏 | 🞏 | 🞏 | 🞏 | 🞏 |
| Now | 🞏 | 🞏 | 🞏 | 🞏 | 🞏 | 🞏 | 🞏 | 🞏 | 🞏 |
| In the future | 🞏 | 🞏 | 🞏 | 🞏 | 🞏 | 🞏 | 🞏 | 🞏 | 🞏 |

| **Helpfulness** (working for the welfare of others) | Opposed to my values | Not important |  |  | Moderately important |  |  | Very important | Of supreme importance |
| --- | --- | --- | --- | --- | --- | --- | --- | --- | --- |
|  | -1 | 0 | 1 | 2 | 3 | 4 | 5 | 6 | 7 |
| In the past | 🞏 | 🞏 | 🞏 | 🞏 | 🞏 | 🞏 | 🞏 | 🞏 | 🞏 |
| Now | 🞏 | 🞏 | 🞏 | 🞏 | 🞏 | 🞏 | 🞏 | 🞏 | 🞏 |
| In the future | 🞏 | 🞏 | 🞏 | 🞏 | 🞏 | 🞏 | 🞏 | 🞏 | 🞏 |
|  |  |  |  |  |  |  |  |  |  |
| **Equality** (equal opportunity for all) | Opposed to my values | Not important |  |  | Moderately important |  |  | Very important | Of supreme importance |
|  | -1 | 0 | 1 | 2 | 3 | 4 | 5 | 6 | 7 |
| In the past | 🞏 | 🞏 | 🞏 | 🞏 | 🞏 | 🞏 | 🞏 | 🞏 | 🞏 |
| Now | 🞏 | 🞏 | 🞏 | 🞏 | 🞏 | 🞏 | 🞏 | 🞏 | 🞏 |
| In the future | 🞏 | 🞏 | 🞏 | 🞏 | 🞏 | 🞏 | 🞏 | 🞏 | 🞏 |
|  |  |  |  |  |  |  |  |  |  |
| **Responsibility** (acting with others in mind) | Opposed to my values | Not important |  |  | Moderately important |  |  | Very important | Of supreme importance |
|  | -1 | 0 | 1 | 2 | 3 | 4 | 5 | 6 | 7 |
| In the past | 🞏 | 🞏 | 🞏 | 🞏 | 🞏 | 🞏 | 🞏 | 🞏 | 🞏 |
| Now | 🞏 | 🞏 | 🞏 | 🞏 | 🞏 | 🞏 | 🞏 | 🞏 | 🞏 |
| In the future | 🞏 | 🞏 | 🞏 | 🞏 | 🞏 | 🞏 | 🞏 | 🞏 | 🞏 |
|  |  |  |  |  |  |  |  |  |  |
| **Honesty** (genuine, sincere) | Opposed to my values | Not important |  |  | Moderately important |  |  | Very important | Of supreme importance |
|  | -1 | 0 | 1 | 2 | 3 | 4 | 5 | 6 | 7 |
| In the past | 🞏 | 🞏 | 🞏 | 🞏 | 🞏 | 🞏 | 🞏 | 🞏 | 🞏 |
| Now | 🞏 | 🞏 | 🞏 | 🞏 | 🞏 | 🞏 | 🞏 | 🞏 | 🞏 |
| In the future | 🞏 | 🞏 | 🞏 | 🞏 | 🞏 | 🞏 | 🞏 | 🞏 | 🞏 |
|  |  |  |  |  |  |  |  |  |  |
| **Forgiveness** (willing to pardon others) | Opposed to my values | Not important |  |  | Moderately important |  |  | Very important | Of supreme importance |
|  | -1 | 0 | 1 | 2 | 3 | 4 | 5 | 6 | 7 |
| In the past | 🞏 | 🞏 | 🞏 | 🞏 | 🞏 | 🞏 | 🞏 | 🞏 | 🞏 |
| Now | 🞏 | 🞏 | 🞏 | 🞏 | 🞏 | 🞏 | 🞏 | 🞏 | 🞏 |
| In the future | 🞏 | 🞏 | 🞏 | 🞏 | 🞏 | 🞏 | 🞏 | 🞏 | 🞏 |
