# Supplementary material for: Perceptions of values over time and why they matter
Source: J Pers. 2020 Dec 5;89(4):689–705. doi: 10.1111/jopy.12608 (PMC8518993; doi:10.1111/jopy.12608)
Supplement: Supplementary file 2 — Supplementary Material [file JOPY-89-689-s003.docx]

**Appendix B**

Behaviours for Study 4 (no constraint / constraint)

We would like you to rate each activity in three ways:

1) How much time you think you spent doing this activity **in the past** / **10 years ago**.

2) How much time you think you spend doing this activity **now**.

3) How much time you think you will spend doing this activity**in the future / in 10 years' time**.

Taking on lots of commitments (SE; Achievement)

Aiming for promotion in your career (SE; Achievement)

Helping out friends and family (ST; Benevolence)

Supporting charities with my time or money (ST; Benevolence)

Trying out a new hobby (O; Self-direction)

Looking for different perspectives on the news (O; Self-direction)

Participating in local traditions (C; Tradition)

Taking interest in British history (C; Tradition)

Trying to accumulate wealth (SE; Power)

Making decisions on behalf of other people (SE; Power)

Trying to protect the environment (ST; Universalism)

Speaking out when I see others being treated unfairly (ST; Universalism)

Visiting new countries (O; Stimulation)

Buying new foods that you haven’t tried before (O; Stimulation)

Acting in line with social expectations (C; Conformity)

Trying to avoid saying anything controversial (C; Conformity)
