# Supplementary material for: Perceptions of values over time and why they matter
Source: J Pers. 2020 Dec 5;89(4):689–705. doi: 10.1111/jopy.12608 (PMC8518993; doi:10.1111/jopy.12608)
Supplement: Supplementary file 3 — Supplementary Material [file JOPY-89-689-s002.docx]

**Values over time – Supplemental Materials**

# MDS


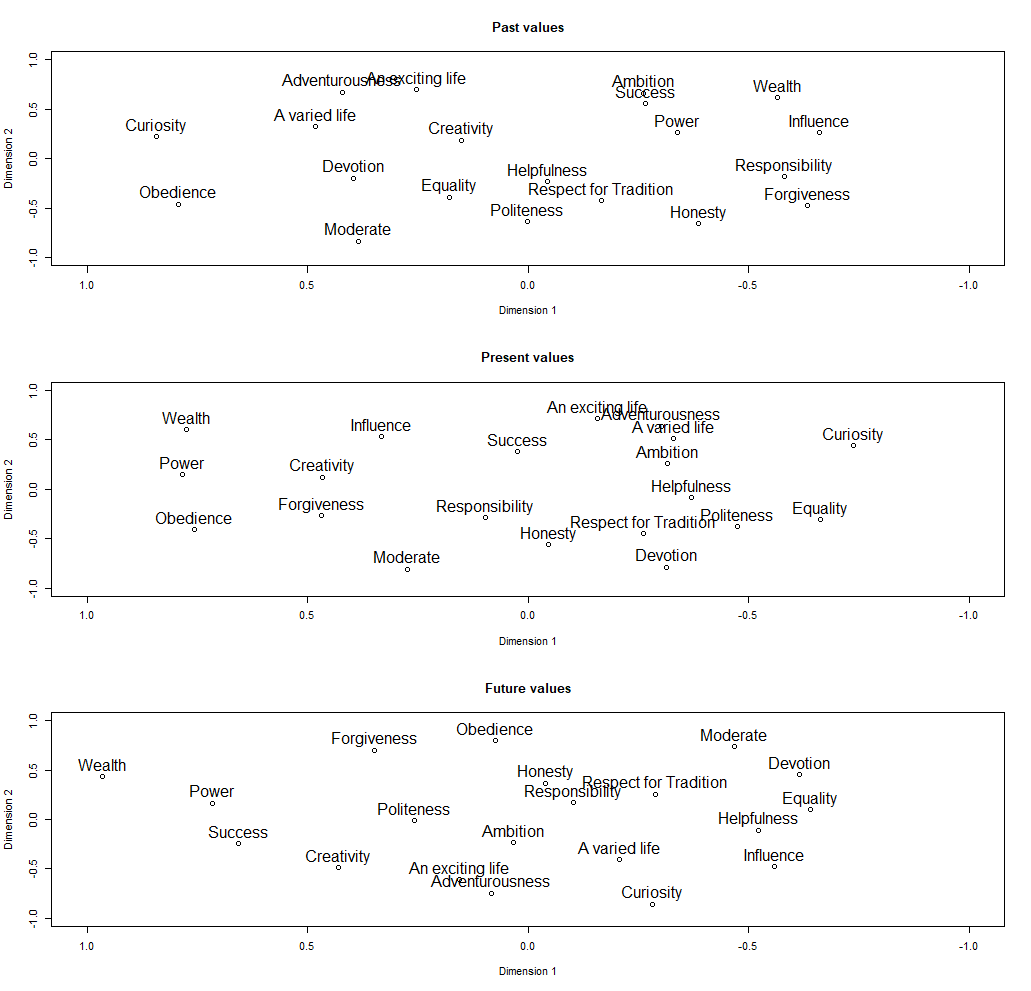


*Figure S1*. Common Space plots of Study 2 (temporal constraint condition). Most self-enhancement (power, wealth, ambition, influence, success), openness (adventurousness, an exciting life, a varied life, creativity, curiosity), self-transcendence (equality, helpfulness, honesty, responsibility, forgiveness), and conservation values (politeness, respect for tradition, obedience, moderate, devotion) are grouped together.


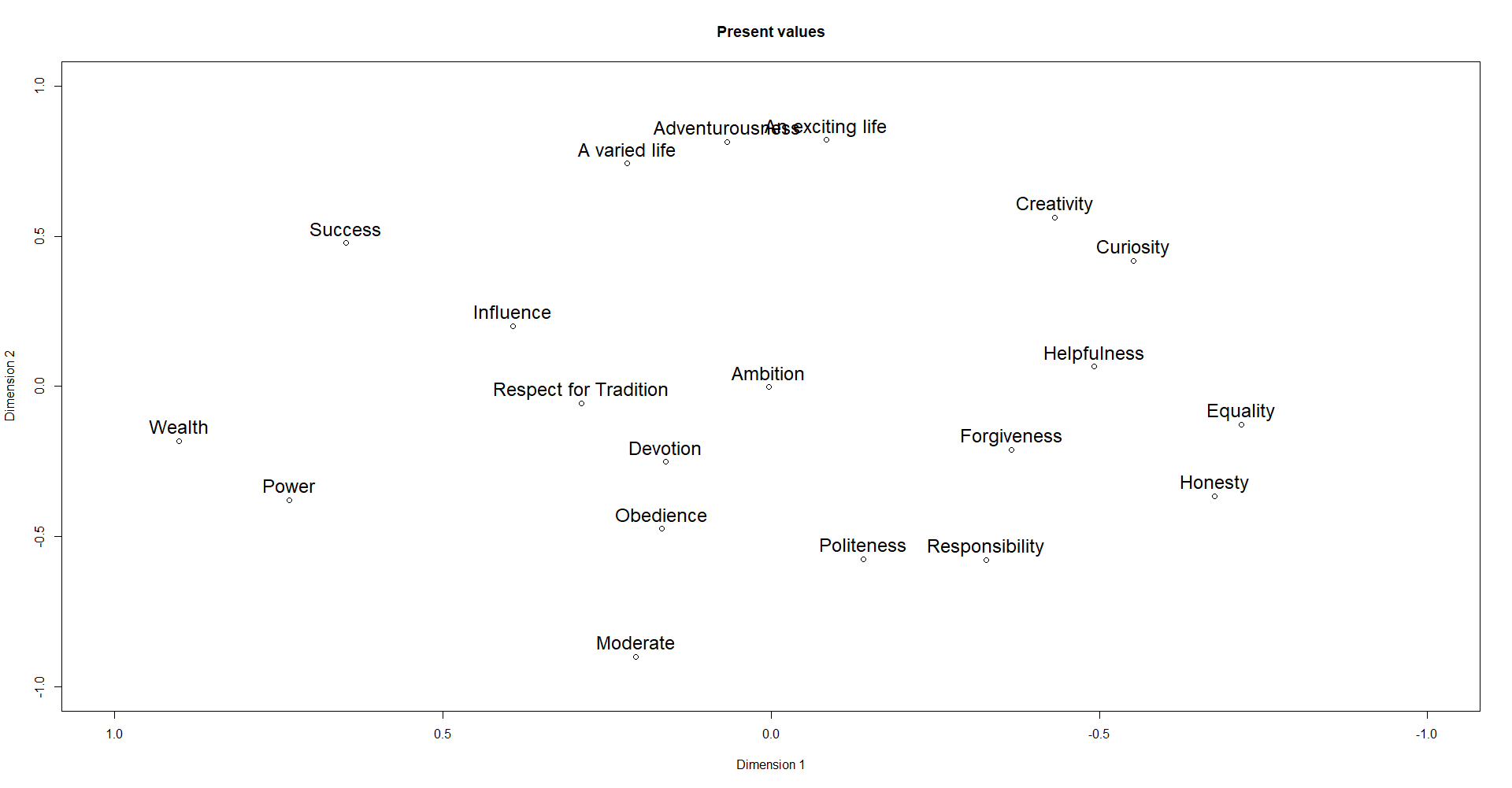


*Figure S2*. Common Space plots of Study 2 (control condition). Most self-enhancement (power, wealth, ambition, influence, success), openness (adventurousness, an exciting life, a varied life, creativity, curiosity), self-transcendence (equality, helpfulness, honesty, responsibility, forgiveness), and conservation values (politeness, respect for tradition, obedience, moderate, devotion) are grouped together.


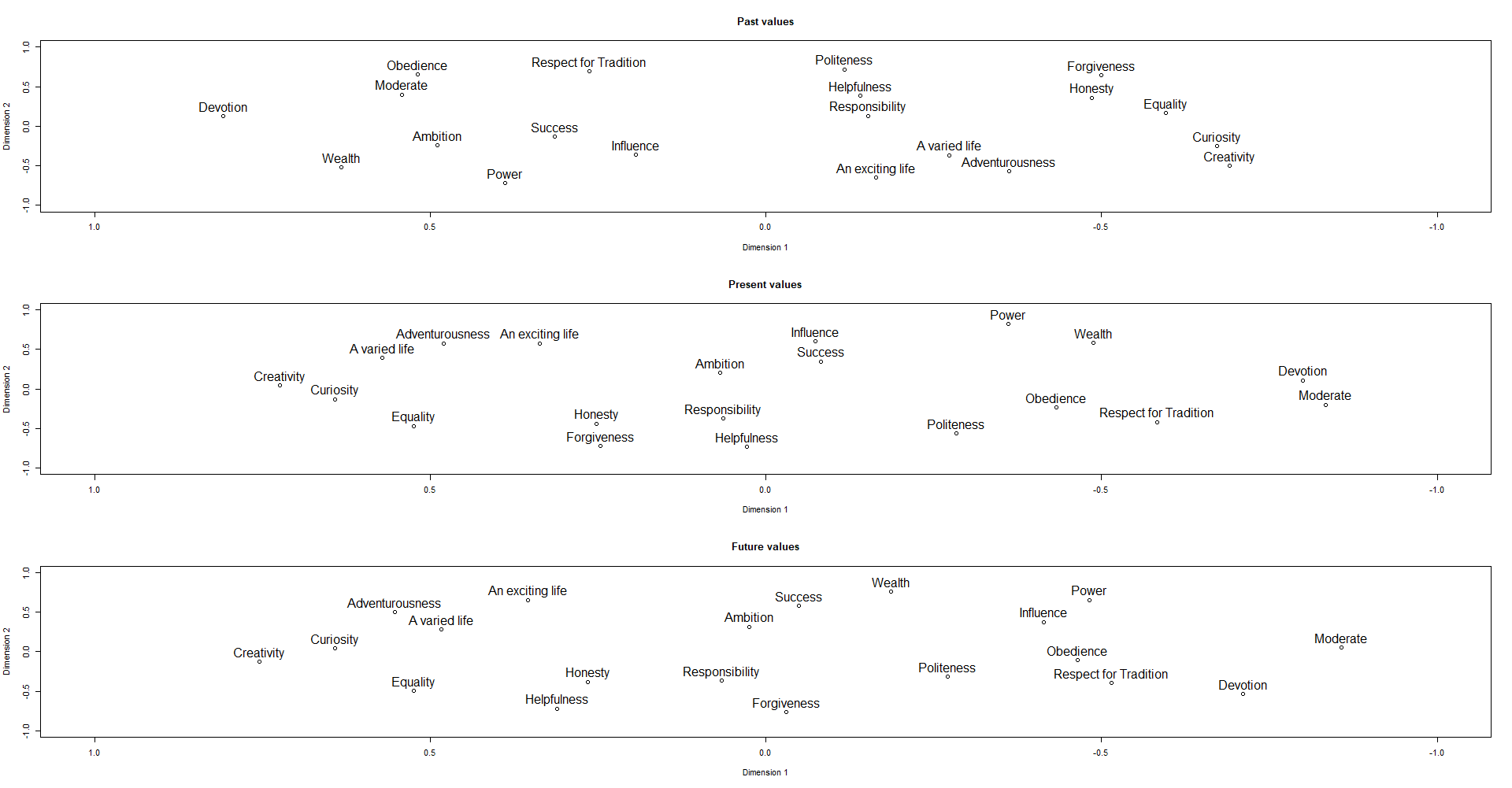


*Figure S3*. Common Space plots of Study 3. Most self-enhancement (power, wealth, ambition, influence, success), openness (adventurousness, an exciting life, a varied life, creativity, curiosity), self-transcendence (equality, helpfulness, honesty, responsibility, forgiveness), and conservation values (politeness, respect for tradition, obedience, moderate, devotion) are grouped together.


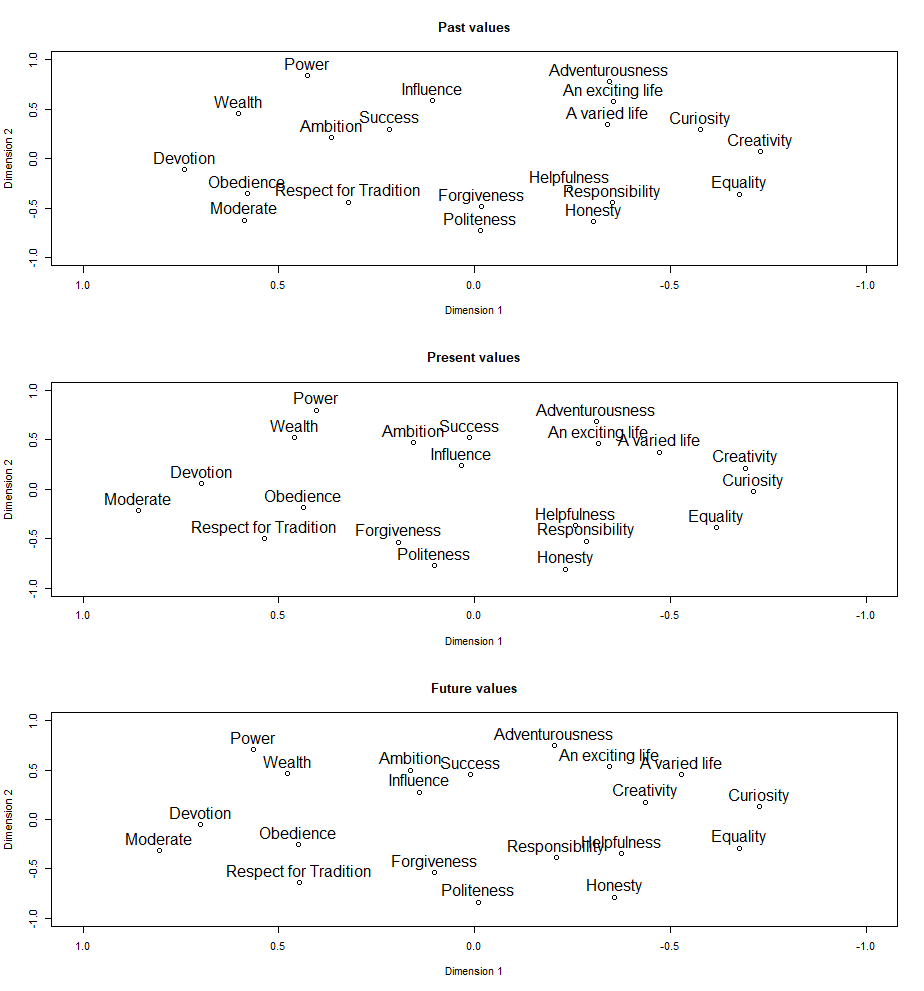


*Figure S4*. Common Space plots of Study 4. All self-enhancement (power, wealth, ambition, influence, success), openness (adventurousness, an exciting life, a varied life, creativity, curiosity), self-transcendence (equality, helpfulness, honesty, responsibility, forgiveness), and conservation values (politeness, respect for tradition, obedience, moderate, devotion) are grouped together.

# Variability in Value Ratings across Time

To test whether people perceived their past self-transcendence, openness, self-enhancement, and conservation to be more similar than their future values, we computed variability first on an individual and then on a group level. We then compared the amount of variability observed in the past condition with the variability in the present condition, and the variability in the present with the one in the future condition. We used the standard deviation to operationalise variability.

Specifically, to operationalise individual variability, we computed the standard deviation across the four higher-order value types, separately for each participant and time point. With two paired *t*-tests we compared then variability past vs present and present vs future. To operationalise group level variability, we computed the means of each higher-order value types, again separately for each time point (as displayed in Figures 2 and 3). To test whether variability differed across time points, we computed a Levene test for homogeneity of variance.

On an individual level, none of the paired t-tests (past vs present and present vs future) for Studies 1-3 was significant, *p*s > .47. For Study 4, however, variability in the past condition (mean SD = 1.12) was on average significantly lower than in the present condition [mean SD = 1.20, *t*(519) = -3.69, *p* < .001]. The variability in the present condition, in turn, was also lower than the variability in the future condition, [mean SD = 1.28, *t*(519) = -6.66, *p* < .001] On the group level, none of the four Levene tests was significant, *p*s > .82.

# Correlations of Value Stability Scores with Well-being (Studies 3 and 4)

| Table S1 |  |  |  |  |  |  |
| --- | --- | --- | --- | --- | --- | --- |
| *Study 3: Correlations between raw and centred value stability scores and well-being* | | | | | | |
|  | SWLS | SPANE Positive | SPANE Negative | SPANE Overall | FS | Well-being Index |
| Self-transcendence (raw) | -.07 | .01 | -.08 | .06 | -.02 | -.02 |
| Openness (raw) | -.09 | -.03 | .02 | -.03 | -.03 | -.07 |
| Self-enhancement (raw) | -.06 | .04 | -.09 | .08 | -.05 | -.02 |
| Conservation (raw) | -.14 | .04 | -.02 | .04 | -.05 | -.06 |
| Self-transcendence (centred) | -.12 | -.03 | -.02 | .00 | -.09 | -.08 |
| Openness (centred) | -.19* | -.02 | .02 | -.03 | -.07 | -.11 |
| Self-enhancement (centred) | -.10 | -.02 | -.03 | .01 | -.05 | -.05 |
| Conservation (centred) | -.11 | -.07 | .03 | -.05 | -.12 | -.11 |
| *Note*. **p*<.05 |  |  |  |  |  |  |

| Table S2 |  |  |  |  |  |  |  |
| --- | --- | --- | --- | --- | --- | --- | --- |
| *Study 4: Correlations between raw and centred value stability scores and well-being* | | | | | | |  |
|  | SWLS | SPANE Positive | SPANE Negative | SPANE Overall | FS | Well-being Index | Optimism |
| Self-transcendence (raw) | -.07 | .02 | .06 | -.02 | .01 | -.02 | -.02 |
| Openness (raw) | -.20*** | -.13** | .11* | -.13** | -.14** | -.16*** | -.11* |
| Self-enhancement (raw) | -.09* | -.06 | .04 | -.05 | -.03 | -.06 | -.09 |
| Conservation (raw) | -.08 | -.01 | .08 | -.05 | -.04 | -.06 | -.02 |
| Self-transcendence (centred) | -.13** | -.06 | .06 | -.06 | -.03 | -.07 | -.08 |
| Openness (centred) | -.15*** | -.10* | .13** | -.13** | -.09* | -.13** | -.08 |
| Self-enhancement (centred) | -.07 | -.03 | .03 | -.03 | .00 | -.03 | -.05 |
| Conservation (centred) | -.10* | -.09* | .09* | -.10* | -.08 | -.10* | -.06 |
| *Note*. **p*<.05, ***p*<.01, ****p* < .001 | | | | | |  |  |

## Mixed-ANOVAs: Interactions for Age and Time (Study 4)

This study’s second aim was to test for age effects. To do this, we performed four mixed-ANOVAs with age group (18-25, 26-35, 36-45, 46-55, and 56-65 years) as the between-subject factor and time (past, present, future) as the within-subject factor. For self-transcendence, we found an interaction, *F*(8, 1030) = 15.23, *p* < .001, *partialη^2^* = .11, a within-subject effect, *F*(2, 1030) = 146.44, *p* < .001, *partialη^2^* = .22, but no between-subject effect, *F*(4, 515) = 0.45, *p* = .77, *partialη^2^* = .00. For openness, we found an interaction, *F*(8, 1028) = 10.31, *p* < .001, *partialη^2^* = .07, no within-subject effect, *F*(2, 1028) = 0.57, *p* = .57, *partialη^2^* = .00, but a between-subject effect, *F*(4, 514) = 6.05, *p* < .001, *partialη^2^* = .04. For self-enhancement, we found an interaction, *F*(8, 1030) = 38.48, *p* < .001, *partialη^2^* = .23, no within-subject effect, *F*(2, 1030) = 0.36, *p* = .70, *partialη^2^* = .00, but a between-subject effect, *F*(4, 515) = 16.58, *p* < .001, *partialη^2^* = .11. For conservation, we found an interaction, *F*(8, 1028) = 15.20, *p* < .001, *partialη^2^* = .11, a within-subject effect, *F*(2, 1028) = 12.66, *p* < .001, *partialη^2^* = .02, and a between-subject effect, *F*(4, 514) = 4.47, *p* = .001, *partialη^2^* = .03.

## Self-reported Behaviours Over Time (Study 4)


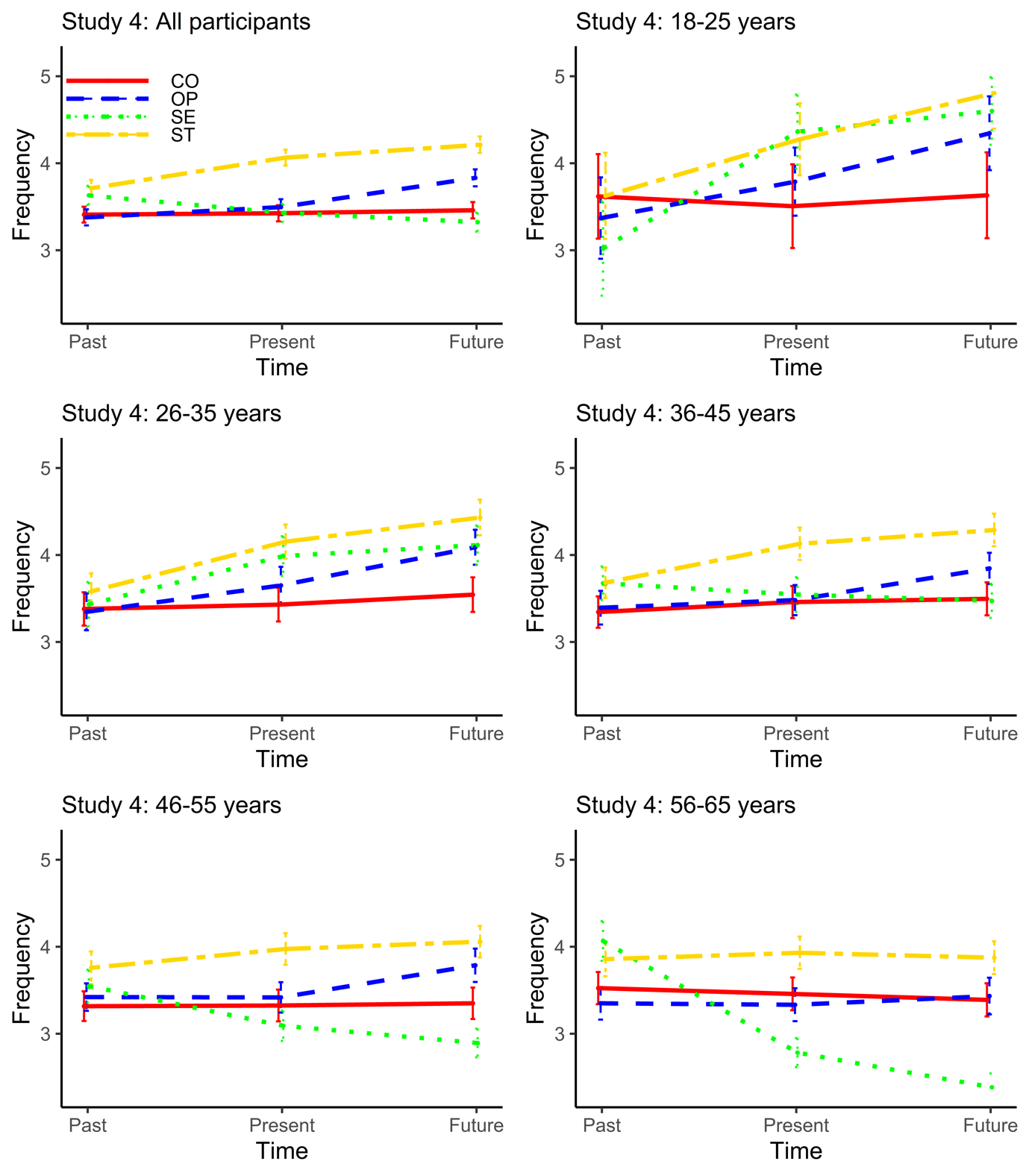


*Figure S5*. Study 4: Self-reported behaviours over time (raw scores).


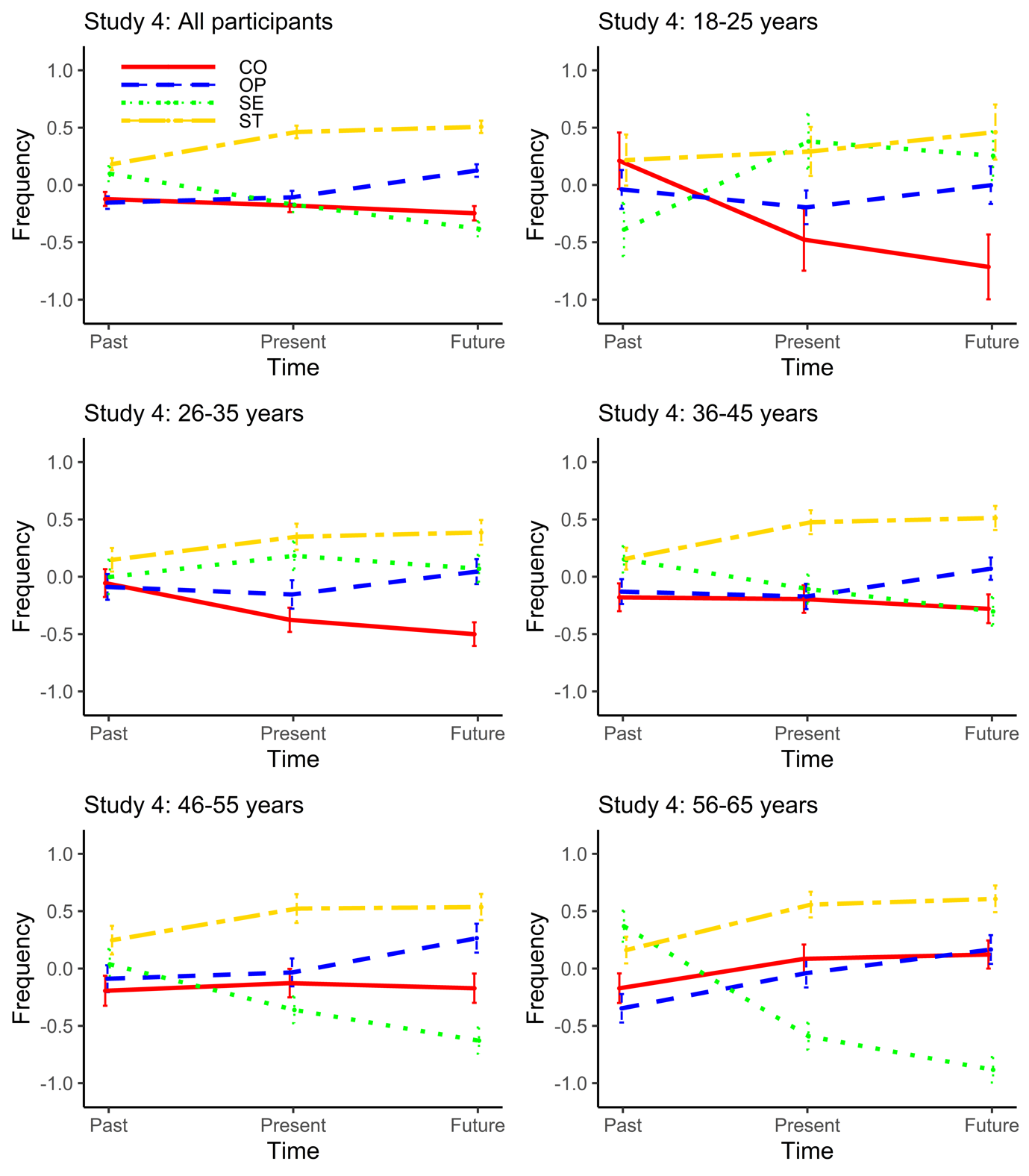
 *Figure S6*. Study 4: Self-reported behaviours over time (centred scores).

**Comparisons of constraining time periods (no constraint vs 10-year constraint; Study 4)**

Table S3

*Study 4: Between-subject comparisons of time constraint vs no time-constraint condition (raw value scores)*

|  | No time constraints | | 10-year constraint | |  |  |  |
| --- | --- | --- | --- | --- | --- | --- | --- |
|  | *M* | *SD* | *M* | *SD* | *t* | *p* | Cohen’s *d* |
| ST past | 6.59 | 1.38 | 6.50 | 1.48 | 0.74 | .46 | 0.06 |
| ST present | 7.10 | 1.15 | 7.05 | 1.20 | 0.49 | .63 | 0.04 |
| ST future | 7.17 | 1.17 | 7.08 | 1.22 | 0.82 | .41 | 0.07 |
| OP past | 6.09 | 1.45 | 6.10 | 1.47 | -0.07 | .94 | -0.01 |
| OP present | 5.92 | 1.42 | 6.00 | 1.39 | -0.63 | .53 | -0.06 |
| OP future | 5.97 | 1.49 | 5.85 | 1.54 | 0.91 | .36 | 0.08 |
| SE past | 5.34 | 1.41 | 5.36 | 1.43 | -0.20 | .84 | -0.02 |
| SE present | 5.03 | 1.51 | 5.27 | 1.49 | -1.79 | .07 | -0.16 |
| SE future | 5.06 | 1.59 | 5.05 | 1.66 | 0.13 | .89 | 0.01 |
| CO past | 5.62 | 1.46 | 5.59 | 1.49 | 0.28 | .78 | 0.02 |
| CO present | 5.58 | 1.38 | 5.68 | 1.41 | -0.79 | .43 | -0.07 |
| CO future | 5.57 | 1.44 | 5.60 | 1.46 | -0.28 | .78 | -0.02 |

*Note*. ST: self-transcendence, OP: openness, SE: self-enhancement, CO: conservation.

Table S4

*Study 4: Between-subject comparisons of time constraint vs no time-constraint condition (centred value scores)*

|  | No time constraints | | 10-year constraint | |  |  |  |
| --- | --- | --- | --- | --- | --- | --- | --- |
|  | *M* | *SD* | *M* | *SD* | *t* | *p* | Cohen’s *d* |
| ST past | 0.68 | 0.89 | 0.61 | 0.98 | 0.85 | .40 | 0.07 |
| ST present | 1.19 | 0.83 | 1.05 | 0.86 | 1.91 | .06 | 0.17 |
| ST future | 1.23 | 0.81 | 1.19 | 0.86 | 0.54 | .59 | 0.05 |
| OP past | 0.18 | 1.01 | 0.21 | 0.99 | -0.37 | .71 | -0.03 |
| OP present | 0.01 | 0.89 | 0.00 | 0.86 | 0.16 | .87 | 0.01 |
| OP future | 0.03 | 0.93 | -0.04 | 0.97 | 0.87 | .39 | 0.08 |
| SE past | -0.57 | 0.99 | -0.52 | 0.96 | -0.56 | .58 | -0.05 |
| SE present | -0.88 | 0.93 | -0.73 | 0.93 | -1.76 | .08 | -0.15 |
| SE future | -0.88 | 0.95 | -0.85 | 1.03 | -0.32 | .75 | -0.03 |
| CO past | -0.29 | 1.03 | -0.30 | 0.95 | 0.15 | .88 | 0.01 |
| CO present | -0.33 | 0.94 | -0.32 | 0.95 | -0.08 | .93 | -0.01 |
| CO future | -0.38 | 1.00 | -0.29 | 1.02 | -0.94 | .35 | -0.08 |

*Note*. ST: self-transcendence, OP: openness, SE: self-enhancement, CO: conservation.

**Within-subject analyses for values over time by age group (Study 4)**

Table S5

*Study 4: Within-subjects linear and quadratic contrasts, separate by values and age group (raw scores)*

| Value | Age | Past | | Present | | Future | | Linear contrasts | | | Quadratic contrasts | | |
| --- | --- | --- | --- | --- | --- | --- | --- | --- | --- | --- | --- | --- | --- |
|  |  | *M* | *SD* | *M* | *SD* | *M* | *SD* | *F* | *p* | *η^2^* | *F* | *p* | *η^2^* |
| ST | 18-25 | 4.99 | 1.84 | 5.37 | 1.15 | 5.58 | 1.03 | 36.36 | <.001 | .48 | 20.20 | <.001 | .34 |
|  | 26-35 | 4.31 | 1.45 | 5.11 | 1.12 | 5.27 | 1.13 | 71.73 | <.001 | .38 | 27.09 | <.001 | .19 |
|  | 36-45 | 4.46 | 1.49 | 5.04 | 1.27 | 5.08 | 1.31 | 31.29 | <.001 | .21 | 30.40 | <.001 | .20 |
|  | 46-55 | 4.60 | 1.31 | 4.99 | 1.20 | 4.99 | 1.22 | 13.95 | <.001 | .11 | 17.94 | <.001 | .13 |
|  | 56-65 | 4.99 | 1.20 | 5.06 | 1.12 | 5.02 | 1.13 | 0.20 | .656 | .00 | 1.45 | .232 | .01 |
| OP | 18-25 | 4.02 | 1.74 | 4.86 | 1.44 | 4.79 | 1.62 | 9.16 | .004 | .19 | 13.42 | .001 | .26 |
|  | 26-35 | 4.15 | 1.48 | 4.28 | 1.31 | 4.31 | 1.34 | 1.67 | .201 | .01 | 0.73 | .396 | .01 |
|  | 36-45 | 4.07 | 1.53 | 3.97 | 1.39 | 3.95 | 1.43 | 0.84 | .365 | .00 | 0.35 | .556 | .00 |
|  | 46-55 | 4.10 | 1.31 | 3.81 | 1.31 | 3.81 | 1.45 | 5.56 | .020 | .05 | 4.95 | .028 | .04 |
|  | 56-65 | 4.08 | 1.43 | 3.49 | 1.38 | 3.30 | 1.52 | 30.45 | <.001 | .20 | 7.87 | .006 | .06 |
| SE | 18-25 | 3.09 | 1.73 | 4.36 | 1.28 | 4.69 | 1.27 | 41.50 | <.001 | .52 | 17.20 | <.001 | .31 |
|  | 26-35 | 3.42 | 1.47 | 3.78 | 1.41 | 3.89 | 1.45 | 10.52 | .002 | .08 | 3.23 | .082 | .03 |
|  | 36-45 | 3.40 | 1.37 | 3.12 | 1.41 | 3.08 | 1.43 | 6.37 | .013 | .05 | 3.10 | .078 | .03 |
|  | 46-55 | 3.13 | 1.28 | 2.84 | 1.37 | 2.61 | 1.49 | 19.75 | <.001 | .14 | 0.21 | .649 | .00 |
|  | 56-65 | 3.54 | 1.42 | 2.46 | 1.40 | 2.10 | 1.36 | 154.81 | <.001 | .57 | 31.28 | <.001 | .21 |
| CO | 18-25 | 3.79 | 1.83 | 3.88 | 1.72 | 3.84 | 1.73 | 0.61 | .809 | .00 | 0.49 | .494 | .01 |
|  | 26-35 | 3.60 | 1.52 | 3.80 | 1.40 | 3.77 | 1.50 | 3.01 | .085 | .03 | 6.89 | .010 | .06 |
|  | 36-45 | 3.36 | 1.54 | 3.49 | 1.48 | 3.51 | 1.52 | 2.51 | .115 | .02 | 3.42 | .067 | .03 |
|  | 46-55 | 3.47 | 1.36 | 3.48 | 1.36 | 3.43 | 1.44 | 0.26 | .611 | .00 | 0.69 | .410 | .01 |
|  | 56-65 | 3.94 | 1.28 | 3.68 | 1.18 | 3.57 | 1.21 | 16.22 | <.001 | .12 | 2.65 | .106 | .02 |

*Note*. Age: Age cohort. ST: self-transcendence, OP: openness, SE: self-enhancement, CO: conservation.

Table S6

*Study 4: Within-subjects linear and quadratic contrasts, separate by values and age group (centred scores)*

| Value | Age | Past | | Present | | Future | | Linear contrasts | | | Quadratic contrasts | | |
| --- | --- | --- | --- | --- | --- | --- | --- | --- | --- | --- | --- | --- | --- |
|  |  | *M* | *SD* | *M* | *SD* | *M* | *SD* | *F* | *p* | *η^2^* | *F* | *p* | *η^2^* |
| ST | 18-25 | 0.27 | 0.75 | 0.76 | 0.69 | 0.86 | 0.70 | 12.91 | <.001 | .25 | 5.38 | .03 | .12 |
|  | 26-35 | 0.44 | 0.96 | 0.87 | 0.74 | 0.96 | 0.71 | 38.87 | <.001 | .25 | 12.62 | <.001 | .10 |
|  | 36-45 | 0.63 | 0.95 | 1.13 | 0.79 | 1.18 | 0.78 | 17.65 | <.001 | .25 | 25.04 | <.001 | .17 |
|  | 46-55 | 0.78 | 0.95 | 1.21 | 0.92 | 1.28 | 0.89 | 46.39 | <.001 | .28 | 23.69 | <.001 | .17 |
|  | 56-65 | 0.85 | 0.88 | 1.38 | 0.88 | 1.52 | 0.87 | 107.50 | <.001 | .47 | 32.76 | <.001 | .22 |
| OP | 18-25 | 0.30 | 1.02 | 0.24 | 0.94 | 0.07 | 1.05 | 1.07 | .27 | .03 | 0.37 | .55 | .01 |
|  | 26-35 | 0.28 | 1.00 | 0.04 | 0.80 | -0.00 | 0.82 | 8.60 | .004 | .07 | 3.86 | .052 | .03 |
|  | 36-45 | 0.25 | 1.03 | 0.07 | 0.87 | 0.04 | 0.93 | 4.27 | .041 | .03 | 3.31 | .07 | .03 |
|  | 46-55 | 0.28 | 0.91 | 0.03 | 0.89 | 0.11 | 0.98 | 3.79 | .054 | .03 | 13.02 | <.001 | .10 |
|  | 56-65 | -0.06 | 1.01 | -0.18 | 0.91 | -0.20 | 1.00 | 2.05 | .15 | .02 | 1.14 | .29 | .01 |
| SE | 18-25 | -0.63 | 1.09 | -0.26 | 1.01 | -0.04 | 1.11 | 12.98 | <.001 | .25 | 0.78 | .38 | .02 |
|  | 26-35 | -0.45 | 1.09 | -0.46 | 0.77 | -0.42 | 0.80 | 0.07 | .79 | .00 | 0.26 | .61 | .00 |
|  | 36-45 | -0.42 | 0.96 | -0.78 | 0.85 | -0.82 | 0.86 | 20.56 | <.001 | .15 | 10.69 | .001 | .08 |
|  | 46-55 | -0.69 | 0.88 | -0.94 | 0.96 | -1.10 | 0.98 | 28.51 | <.001 | .19 | 1.05 | .31 | .01 |
|  | 56-65 | -0.60 | 0.91 | -1.21 | 0.92 | -1.40 | 0.89 | 104.31 | <.001 | .47 | 25.67 | <.001 | .18 |
| CO | 18-25 | 0.06 | 1.06 | -0.74 | 1.12 | -0.89 | 1.08 | 23.40 | <.001 | .37 | 10.76 | .002 | .22 |
|  | 26-35 | -0.27 | 0.97 | -0.45 | 0.84 | -0.54 | 0.92 | 10.98 | .001 | .09 | 1.44 | .23 | .01 |
|  | 36-45 | -0.46 | 1.06 | -0.42 | 0.95 | -0.40 | 0.98 | 0.81 | .37 | .01 | 0.21 | .65 | .00 |
|  | 46-55 | -0.36 | 0.94 | -0.30 | 0.96 | -0.28 | 1.03 | 1.04 | .31 | .01 | 0.29 | .59 | .00 |
|  | 56-65 | -0.20 | 0.94 | 0.01 | 0.86 | 0.07 | 0.93 | 11.54 | <.001 | .09 | 4.75 | .031 | .04 |

*Note*. Age: Age cohort. ST: self-transcendence, OP: openness, SE: self-enhancement, CO: conservation.

**Correlations between Values Over Time, Well-being, and Political Orientation (Study 4)**

Table S7

*Study 4: Correlations between raw values data, raw behaviour, well-being, and political orientation.*

|  | α | STb past | STb present | STb future | OPb past | OPb present | OPb future | SEb past | SEb present | SEb future | COb past | COb present | COb future | SWLS | SPANE+ | SPANE - | SPANE Overall | FS | WB-Index | Opt | PO |
| --- | --- | --- | --- | --- | --- | --- | --- | --- | --- | --- | --- | --- | --- | --- | --- | --- | --- | --- | --- | --- | --- |
| α |  | .68 | .69 | .72 | .60 | .60 | .68 | .70 | .71 | .79 | .53 | .62 | .61 | .90 | .91 | .87 | .92 | .88 | .95 | .87 |  |
| ST past | .81 | **.51** | **.29** | **.25** | **.26** | **.12** | **.10** | **.34** | -.02 | -.06 | **.33** | **.19** | **.18** | **.11** | **.14** | -.06 | **.11** | **.13** | **.13** | **.10** | -.09 |
| ST present | .77 | **.38** | **.52** | **.52** | **.21** | **.28** | **.30** | **.24** | **.26** | **.23** | **.29** | **.32** | **.31** | **.18** | **.25** | -.05 | **.16** | **.31** | **.24** | **.16** | -.09 |
| ST future | .77 | **.35** | **.50** | **.53** | **.20** | **.28** | **.32** | **.19** | **.27** | **.29** | **.25** | **.29** | **.31** | **.15** | **.24** | -.04 | **.15** | **.26** | **.21** | **.14** | -.09 |
| OP past | .78 | **.41** | **.35** | **.33** | **.48** | **.35** | **.31** | **.38** | **.23** | **.20** | **.24** | **.24** | **.23** | **.10** | **.16** | -.06 | **.12** | **.18** | **.15** | **.09** | .03 |
| OP present | .79 | **.27** | **.40** | **.44** | **.28** | **.53** | **.53** | **.21** | **.43** | **.44** | **.22** | **.27** | **.29** | **.17** | **.32** | -.08 | **.22** | **.29** | **.26** | **.23** | .04 |
| OP future | .81 | **.28** | **.39** | **.46** | **.27** | **.50** | **.57** | **.16** | **.41** | **.48** | **.21** | **.26** | **.30** | **.13** | **.28** | -.03 | **.17** | **.25** | **.21** | **.19** | -.01 |
| SE past | .76 | **.29** | **.24** | **.21** | **.30** | **.25** | **.21** | **.56** | **.25** | **.16** | **.28** | **.29** | **.26** | .05 | **.14** | -.05 | **.10** | **.12** | **.11** | .08 | .10 |
| SE present | .82 | **.21** | **.33** | **.39** | **.23** | **.39** | **.41** | **.21** | **.62** | **.58** | **.26** | **.32** | **.32** | .**10** | **.21** | .05 | .08 | **.18** | **.14** | **.14** | .11 |
| SE future | .82 | **.16** | **.30** | **.40** | **.20** | **.35** | **.42** | **.10** | **.59** | **.67** | **.23** | **.29** | **.32** | .05 | **.17** | .08 | .05 | **.14** | **.09** | **.09** | .05 |
| CO past | .74 | **.42** | **.29** | **.29** | **.26** | **.22** | **.19** | **.32** | **.16** | **.15** | **.57** | **.48** | **.49** | .04 | **.11** | -.01 | .06 | .08 | .07 | .06 | **.20** |
| CO present | .71 | **.39** | **.40** | **.41** | **.25** | **.28** | **.26** | **.27** | **.31** | **.28** | **.51** | **.58** | **.58** | .08 | **.18** | .01 | **.09** | **.14** | **.12** | .08 | **.24** |
| CO future | .72 | **.36** | **.39** | **.41** | **.24** | **.26** | **.25** | **.21** | **.29** | **.29** | **.46** | **.54** | **.56** | .06 | **.17** | .02 | .08 | **.13** | **.11** | .06 | **.24** |

*Note.* α = Cronbach’s alpha (internal consistency), ST: self-transcendence, OP: openness, SE: self-enhancement, CO: conservation, b: behaviour, SWLS: Satisfaction with Life Scale, SPANE: Scale of Positive and Negative Experience, FS: Flourishing Scale, WB: well-being, Opt: Optimism, PO: Political Orientation. Significant correlations are in bold. All |*r*s| > .08 significant at *p* < .05, all |*r*s| > .11 significant at *p* < .01, |*r*s| > .14 significant at *p* < .001 (*N* = 519-520). For correlations with PO: All |*r*s| > .10 significant at *p* < .05, all |*r*s| > .13 significant at *p* < .01, |*r*s| > .17 significant at *p* < .001 (*N* = 359)

Table S8

*Study 4: Correlations between centred values data, centred behaviour, well-being, and political orientation.*

|  | α | STb past | STb present | STb future | OPb past | OPb present | OPb future | SEb past | SEb present | SEb future | COb past | COb present | COb future | SWLS | SPANE+ | SPANE - | SPANE Overall | FS | WB-Index | Opt | PO |
| --- | --- | --- | --- | --- | --- | --- | --- | --- | --- | --- | --- | --- | --- | --- | --- | --- | --- | --- | --- | --- | --- |
| α |  | .68 | .69 | .72 | .60 | .60 | .68 | .70 | .71 | .79 | .53 | .62 | .61 | .90 | .91 | .87 | .92 | .88 | .95 | .87 |  |
| ST past | .81 | **.33** | **.26** | **.23** | **-.14** | -.02 | .00 | **-.11** | **-.19** | **-.19** | -.03 | -.02 | .00 | .06 | -.00 | -.02 | .01 | .00 | .02 | .02 | **-.23** |
| ST present | .77 | **.16** | **.38** | **.34** | -.08 | -.08 | -.03 | .01 | **-.27** | **-.26** | -.08 | .01 | .00 | .04 | -.04 | -.04 | .00 | .06 | .03 | -.02 | **-.25** |
| ST future | .77 | **.13** | **.36** | **.35** | -.09 | -.06 | -.03 | .07 | **-.25** | **-.30** | **-.11** | -.01 | .03 | .05 | -.03 | -.07 | .02 | .04 | .04 | -.01 | **-.22** |
| OP past | .78 | -.01 | .03 | .02 | **.37** | **.15** | **.16** | -.06 | .08 | .09 | **-.26** | **-.25** | **-.25** | .04 | .04 | -.03 | .04 | .08 | .06 | .02 | -.05 |
| OP present | .79 | -.03 | -.06 | -.07 | **.18** | **.36** | **.33** | .02 | .01 | .06 | **-.16** | **-.29** | **-.30** | .07 | **.14** | **-.11** | **.13** | **.11** | **.13** | **.14** | -.06 |
| OP future | .81 | .01 | -.04 | -.07 | **.14** | **.32** | **.39** | .02 | -.02 | .00 | **-.16** | **-.25** | **-.28** | .06 | **.12** | -.07 | **.11** | **.11** | **.11** | **.12** | **-.09** |
| SE past | .76 | **-.30** | **-.17** | **-.14** | -.04 | .01 | .04 | **.40** | **.19** | **.13** | **-.14** | -.06 | -.05 | -.04 | .00 | .00 | .00 | -.01 | -.01 | .00 | .06 |
| SE present | .82 | **-.14** | **-.29** | **-.23** | .09 | -.04 | -.02 | .06 | **.47** | **.42** | -.03 | **-.20** | **-.22** | -.04 | -.02 | **.10** | -.07 | -.04 | -.05 | 00 | .06 |
| SE future | .82 | **-.14** | **-.28** | **-.26** | **.11** | -.05 | -.08 | -.03 | **.47** | **.54** | .05 | **-.20** | **-.26** | -.06 | -.03 | **.11** | -.08 | -.05 | -.07 | -.02 | .01 |
| CO past | .74 | .00 | **-.11** | **-.10** | **-.20** | **-.14** | **-.20** | **-.23** | -.08 | -.04 | **.43** | **.32** | **.30** | -.05 | -.03 | .05 | -.05 | -.07 | -.06 | -.04 | .**20** |
| CO present | .71 | .02 | .00 | -.02 | **-.19** | **-.23** | **-.26** | -.08 | **-.24** | **-.24** | **.25** | **.47** | **.50** | -.07 | -.08 | .04 | -.07 | **-.12** | **-.09** | **-.10** | **.24** |
| CO future | .72 | .01 | .02 | .03 | **-.17** | **-.20** | **-.26** | -.04 | **-.24** | **-.28** | **.19** | **.44** | **.50** | -.05 | -.06 | .01 | -.04 | **-.09** | -.07 | -.08 | **.26** |

*Note.* α = Cronbach’s alpha (internal consistency), ST: self-transcendence, OP: openness, SE: self-enhancement, CO: conservation, b: behaviour, SWLS: Satisfaction with Life Scale, SPANE: Scale of Positive and Negative Experience, FS: Flourishing Scale, WB: well-being, Opt: Optimism, PO: Political Orientation. Significant correlations are in bold. All |*r*s| > .08 significant at *p* < .05, all |*r*s| > .11 significant at *p* < .01, |*r*s| > .14 significant at *p* < .001 (*N* = 519-520). For correlations with PO: All |*r*s| > .10 significant at *p* < .05, all |*r*s| > .13 significant at *p* < .01, |*r*s| > .17 significant at *p* < .001 (*N* = 359)

Table S9

*Study 4: Correlations between centred values data, raw behaviour, well-being, and political orientation.*

|  | α | STb past | STb present | STb future | OPb past | OPb present | OPb future | SEb past | SEb present | SEb future | COb past | COb present | COb future | SWLS | SPANE+ | SPANE - | SPANE Overall | FS | WB-Index | Opt | PO |
| --- | --- | --- | --- | --- | --- | --- | --- | --- | --- | --- | --- | --- | --- | --- | --- | --- | --- | --- | --- | --- | --- |
| α |  | .68 | .69 | .72 | .60 | .60 | .68 | .70 | .71 | .79 | .53 | .62 | .61 | .90 | .91 | .87 | .92 | .88 | .95 | .87 |  |
| ST past | .81 | .**16** | -.01 | -.04 | **-.11** | **-.17** | **-.16** | **-.09** | **-.26** | **-.26** | -.04 | **-.17** | **-.17** | .06 | -.00 | -.02 | .01 | .00 | .02 | .02 | **-.23** |
| ST present | .77 | .04 | .**07** | .02 | -.1 | **-.21** | **-.19** | -.04 | **-.31** | **-.31** | **-.11** | **-.15** | **-.18** | .04 | -.04 | -.04 | .00 | .06 | .03 | -.02 | **-.25** |
| ST future | .77 | .02 | .05 | -.01 | **-.11** | **-.20** | **-.22** | -.01 | **-.31** | **-.36** | **-.13** | **-.18** | **-.19** | .05 | -.03 | -.07 | .02 | .04 | .04 | -.01 | **-.22** |
| OP past | .78 | .01 | .**09** | .**09** | **.23** | **.17** | **.17** | -.03 | **.11** | **.13** | **-.16** | **-.09** | **-.09** | .04 | .04 | -.03 | .04 | .08 | .06 | .02 | -.05 |
| OP present | .79 | -.05 | .00 | .03 | **.07** | **.26** | **.25** | -.03 | .04 | **.10** | **-.15** | **-.15** | **-.13** | .07 | **.14** | **-.11** | **.13** | **.11** | **.13** | **.14** | -.06 |
| OP future | .81 | .01 | .03 | .05 | **.08** | **.25** | **.30** | .01 | .04 | **.08** | **-.11** | **-.11** | **-.10** | .06 | **.12** | -.07 | **.11** | **.11** | **.11** | **.12** | **-.09** |
| SE past | .76 | **-.19** | **-.09** | **-.10** | -.04 | .02 | .01 | **.23** | **.13** | **.07** | **-.11** | -.02 | -.05 | -.04 | .00 | .00 | .00 | -.01 | -.01 | .00 | .06 |
| SE present | .82 | **-.11** | **-.07** | -.01 | .02 | **.09** | **.11** | .01 | **.39** | **.35** | -.05 | -.02 | -.03 | -.04 | -.02 | **.10** | -.07 | -.04 | -.05 | 00 | .06 |
| SE future | .82 | **-.14** | **-.07** | .01 | .00 | **.07** | **.11** | **-.07** | **.38** | **.46** | -.04 | -.03 | -.01 | -.06 | -.03 | **.11** | -.08 | -.05 | -.07 | -.02 | .01 |
| CO past | .74 | .03 | .00 | .04 | **-.09** | -.02 | -.02 | **-.11** | .01 | .06 | **.32** | **.27** | **.30** | -.05 | -.03 | .05 | -.05 | -.07 | -.06 | -.04 | .**20** |
| CO present | .71 | **.13** | .01 | -.03 | .01 | **-.14** | **-.17** | .06 | **-.14** | **-.16** | **.29** | **.31** | **.31** | -.07 | -.08 | .04 | -.07 | **-.12** | **-.09** | **-.10** | **.24** |
| CO future | .72 | **.12** | .00 | -.05 | .01 | **-.14** | **-.21** | **.07** | **-.16** | **-.23** | **.25** | **.28** | **.27** | -.05 | -.06 | .01 | -.04 | **-.09** | -.07 | -.08 | **.26** |

*Note.* α = Cronbach’s alpha (internal consistency), ST: self-transcendence, OP: openness, SE: self-enhancement, CO: conservation, b: behaviour, SWLS: Satisfaction with Life Scale, SPANE: Scale of Positive and Negative Experience, FS: Flourishing Scale, WB: well-being, Opt: Optimism, PO: Political Orientation. Significant correlations are in bold. All |*r*s| > .08 significant at *p* < .05, all |*r*s| > .11 significant at *p* < .01, |*r*s| > .14 significant at *p* < .001 (*N* = 519-520). For correlations with PO: All |*r*s| > .10 significant at *p* < .05, all |*r*s| > .13 significant at *p* < .01, |*r*s| > .17 significant at *p* < .001 (*N* = 359).

## Moderated regressions between age and value importance with time as moderator (Study 4)

Table S10

*Results of moderated regression (raw value scores)*

|  | Self-transcendence | | | Openness | | | Self-enhancement | | | Conservation | | |
| --- | --- | --- | --- | --- | --- | --- | --- | --- | --- | --- | --- | --- |
|  | *B* | *SE* | *p* | *B* | *SE* | *p* | *B* | *SE* | *p* | *B* | *SE* | *p* |
| Pa-Pr | -1.80 | .29 | <.001 | -1.08 | .33 | .001 | -1.94 | 0.32 | <.001 | -.052 | .33 | .11 |
| Pr-Fu | 0.32 | .29 | .26 | 0.18 | .33 | .58 | 0.66 | .32 | .04 | 0.06 | .33 | .86 |
| Age | -0.01 | .00 | .16 | -0.03 | .01 | <.001 | -0.05 | .00 | <.001 | -0.01 | .01 | .20 |
| Pa-Pr*Age | 0.03 | .01 | <.001 | 0.03 | .01 | <.001 | 0.05 | .01 | <.001 | 0.01 | .01 | .11 |
| Pr-Fu*Age | -0.01 | .01 | .32 | -0.01 | .01 | .46 | -0.02 | .01 | .01 | -0.00 | .01 | .74 |
|  | *F*(5, 1503) *=* 20.34, *R^2^ =* .06*, p* <.001 | | | *F*(5, 1500) = 17.66, *R^2^* = .06, *p* <.001 | | | *F*(5, 1503) = 52.3, *R^2^* = .15, *p* <.001 | | | *F*(5, 1500) = 1.20, *R^2^* = .00, *p* = .31 | | |

*Note.* Pa: Past values, Pr: Present values, Fu: Future values. Present values are used as reference group and compared with past and future values.

Table S11

*Results of moderated regression (centred value scores)*

|  | Self-transcendence | | | Openness | | | Self-enhancement | | | Conservation | | |
| --- | --- | --- | --- | --- | --- | --- | --- | --- | --- | --- | --- | --- |
|  | *B* | *SE* | *p* | *B* | *SE* | *p* | *B* | *SE* | *p* | *B* | *SE* | *p* |
| Pa-Pr | -0.47 | .19 | .001 | 0.26 | .22 | .23 | -0.61 | .21 | .004 | 0.83 | .22 | <.001 |
| Pr-Fu | 0.02 | .19 | .93 | -0.13 | .22 | .55 | 0.36 | .21 | .09 | -0.25 | .22 | .26 |
| Age | 0.02 | .00 | <.001 | -0.01 | .00 | .02 | -0.02 | .00 | <.001 | 0.02 | .00 | <.001 |
| Pa-Pr*Age | -0.00 | .00 | .96 | -0.00 | .00 | .73 | 0.02 | .00 | <.001 | -0.02 | .00 | <.001 |
| Pr-Fu*Age | 0.00 | .00 | .73 | 0.00 | .00 | .58 | -0.01 | .00 | .04 | 0.01 | .00 | .26 |
|  | *F*(5, 1503) = 42.78, *R^2^* = .12, *p* <.001 | | | *F*(5, 1500) = 6.03, *R^2^* = .02, *p* <.001 | | | *F*(5, 1503) = 38.44, *R^2^* = .11, *p* <.001 | | | *F*(5, 1500) = 11.99, *R^2^* = .04, *p* <. 001 | | |

*Note.* Pa: Past values, Pr: Present values, Fu: Future values. Present values are used as reference group and compared with past and future values.


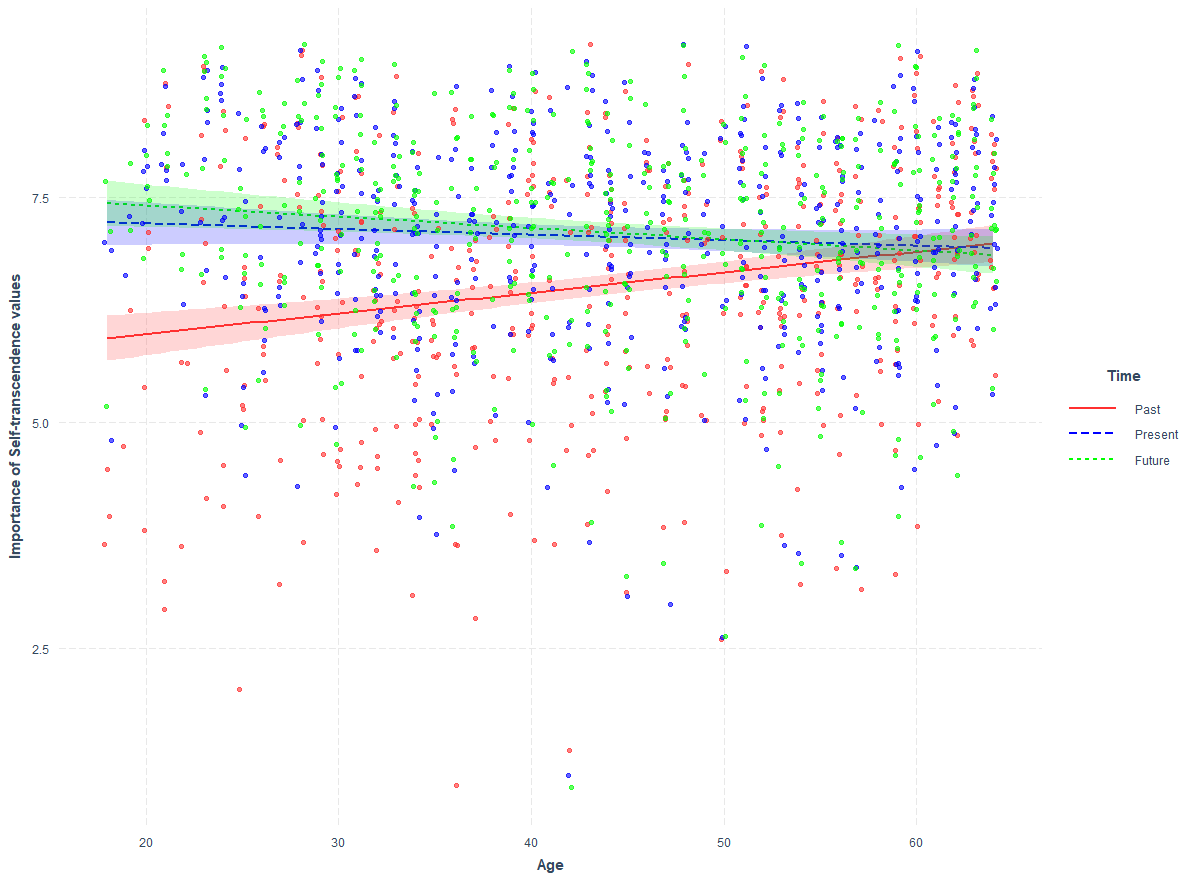


*Figure S7*. Interaction plot of age predicting raw self-transcendence values with time as a moderator.


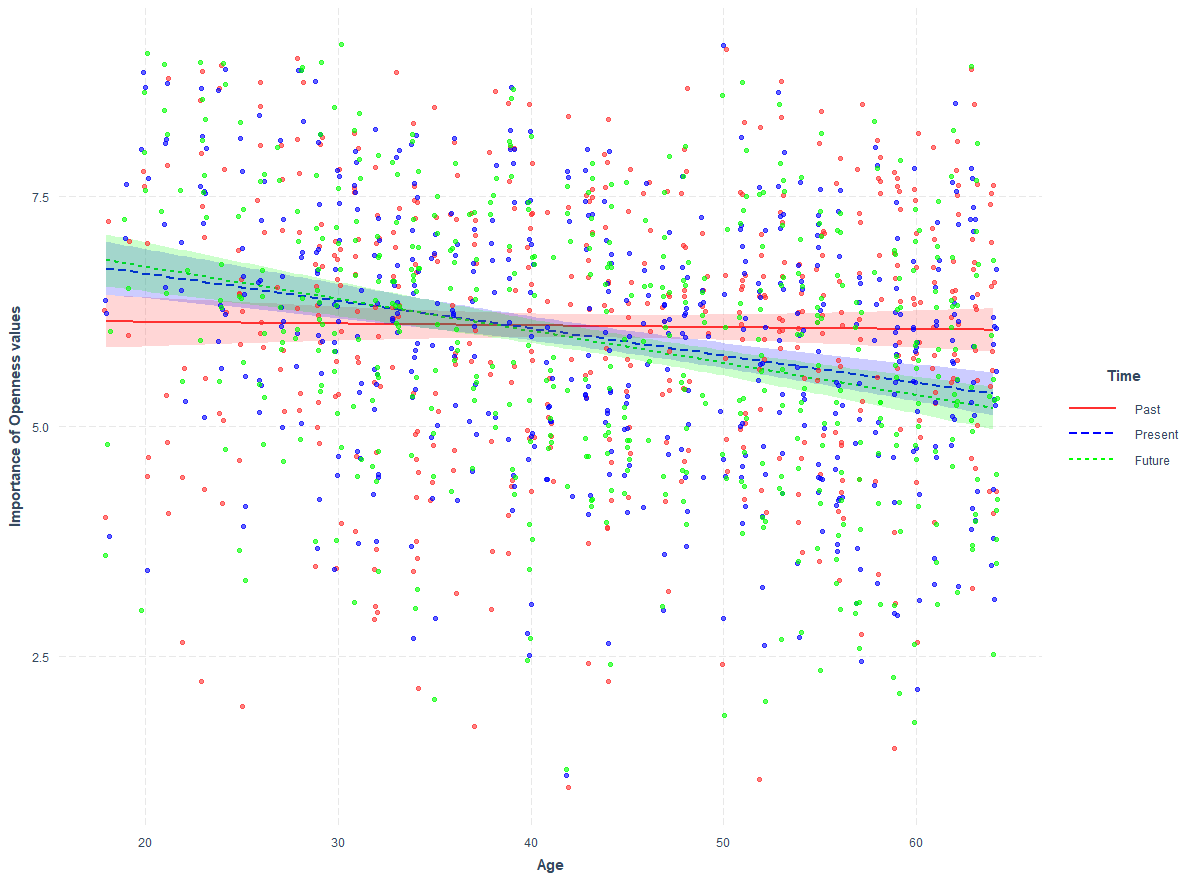


*Figure S8*. Interaction plot of age predicting raw openness values with time as a moderator.


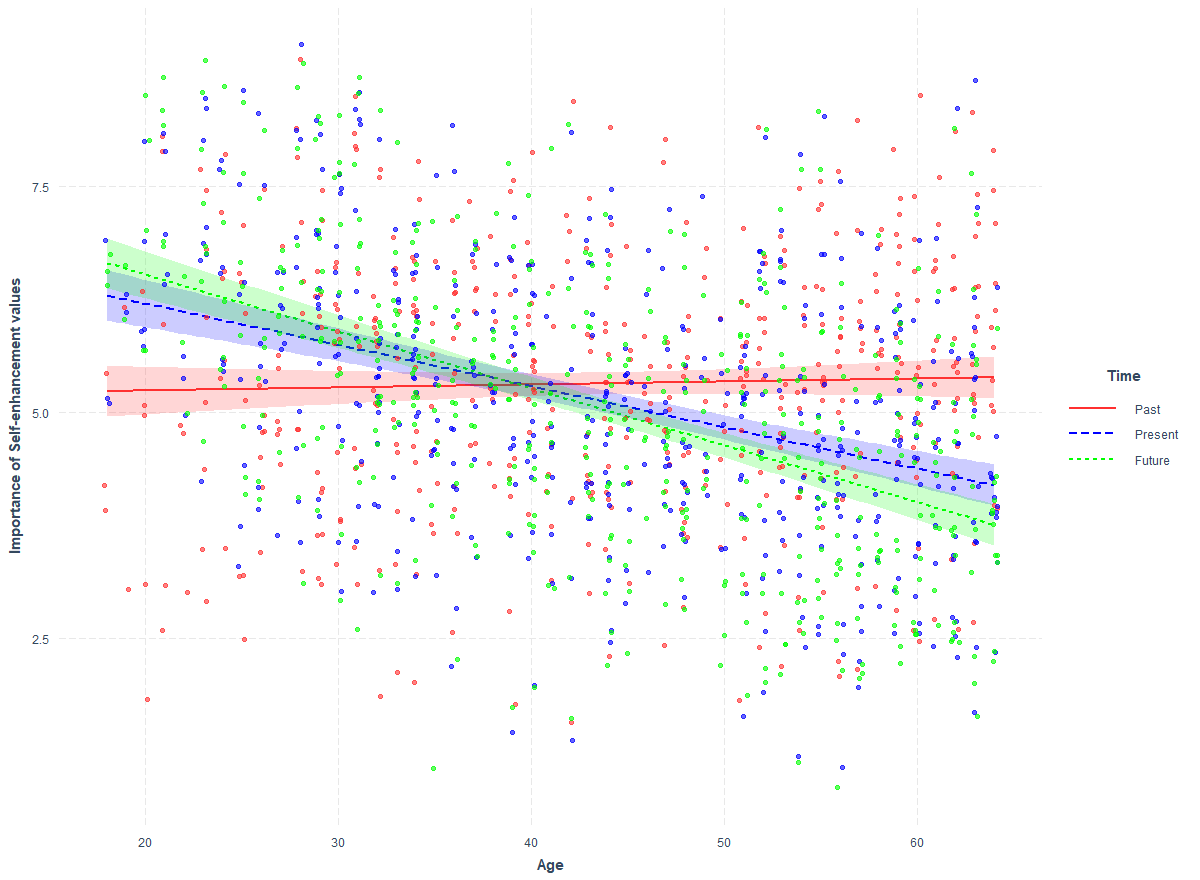


*Figure S9*. Interaction plot of age predicting raw self-enhancement values with time as a moderator.


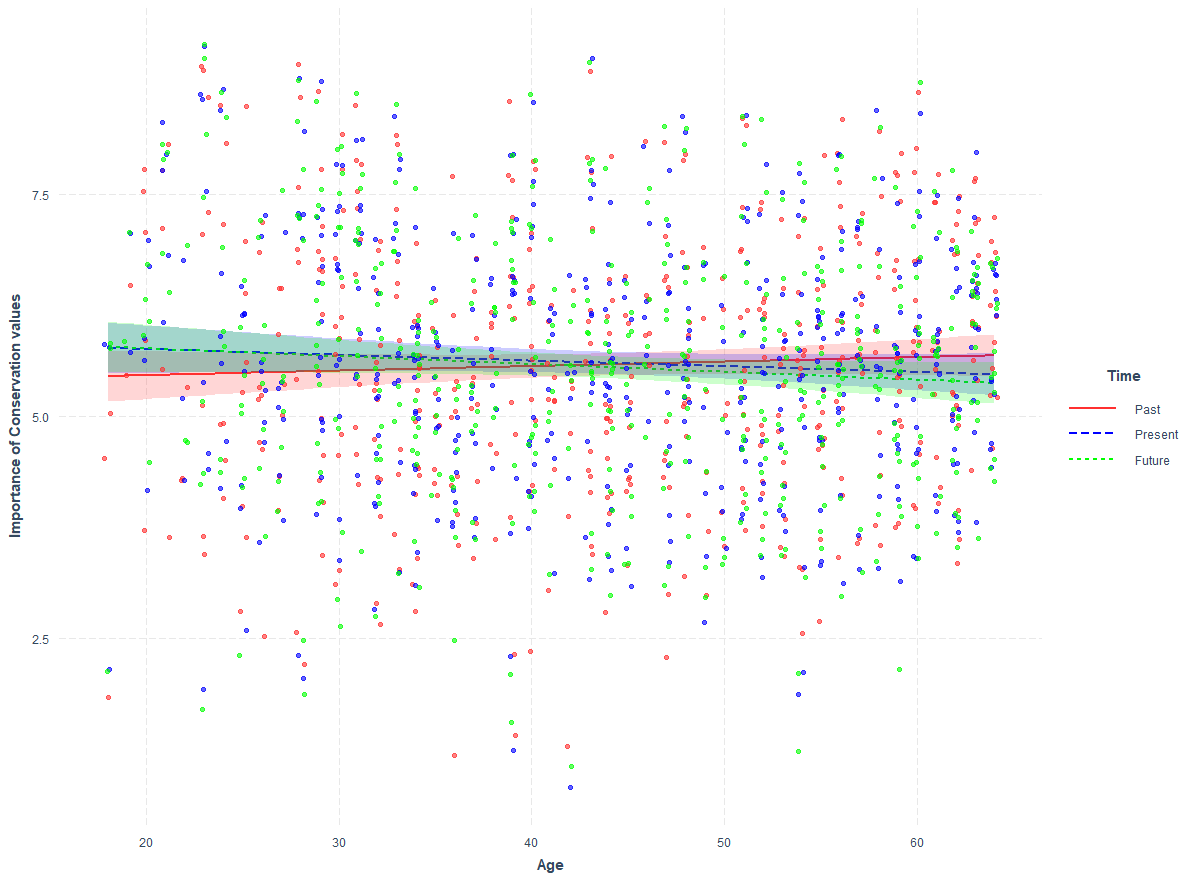


*Figure S10*. Interaction plot of age predicting raw conservation values with time as a moderator.


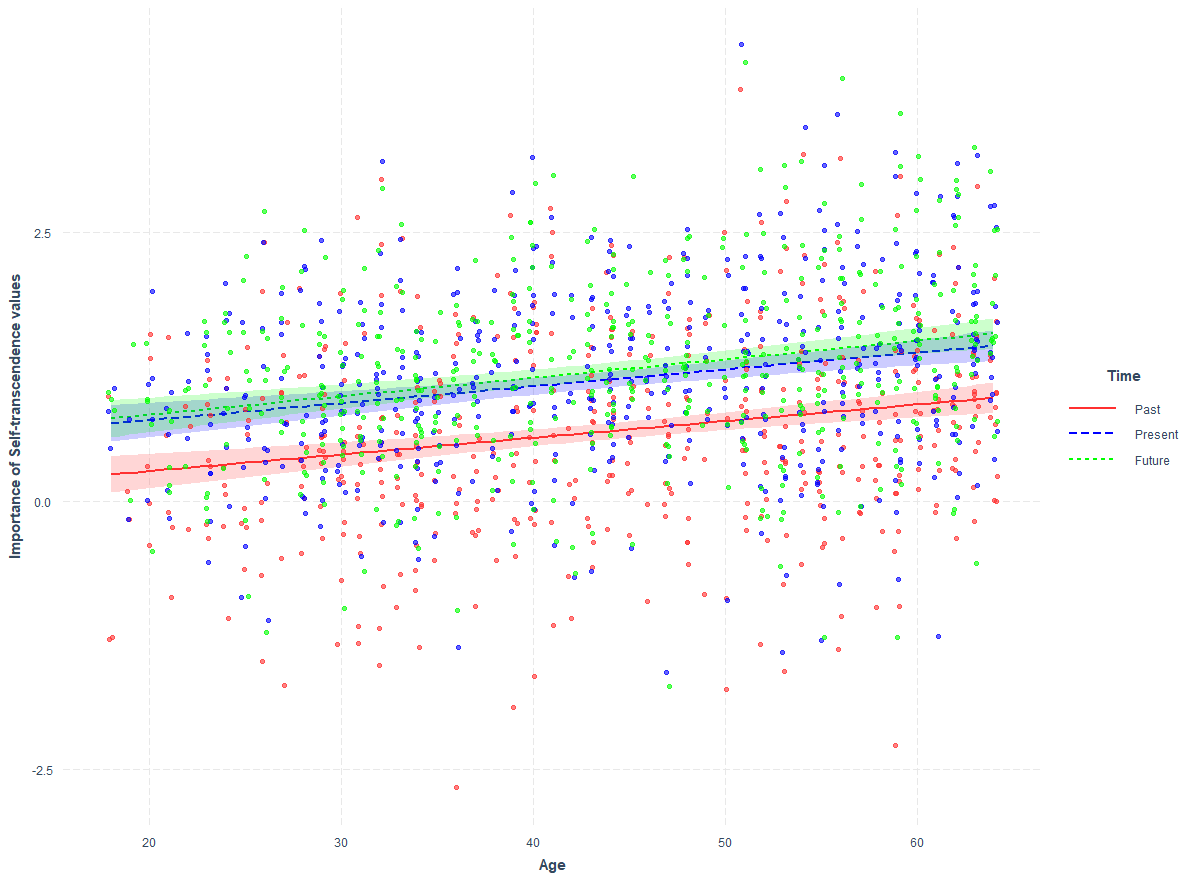


*Figure S11*. Interaction plot of age predicting centred self-transcendence values with time as a moderator.


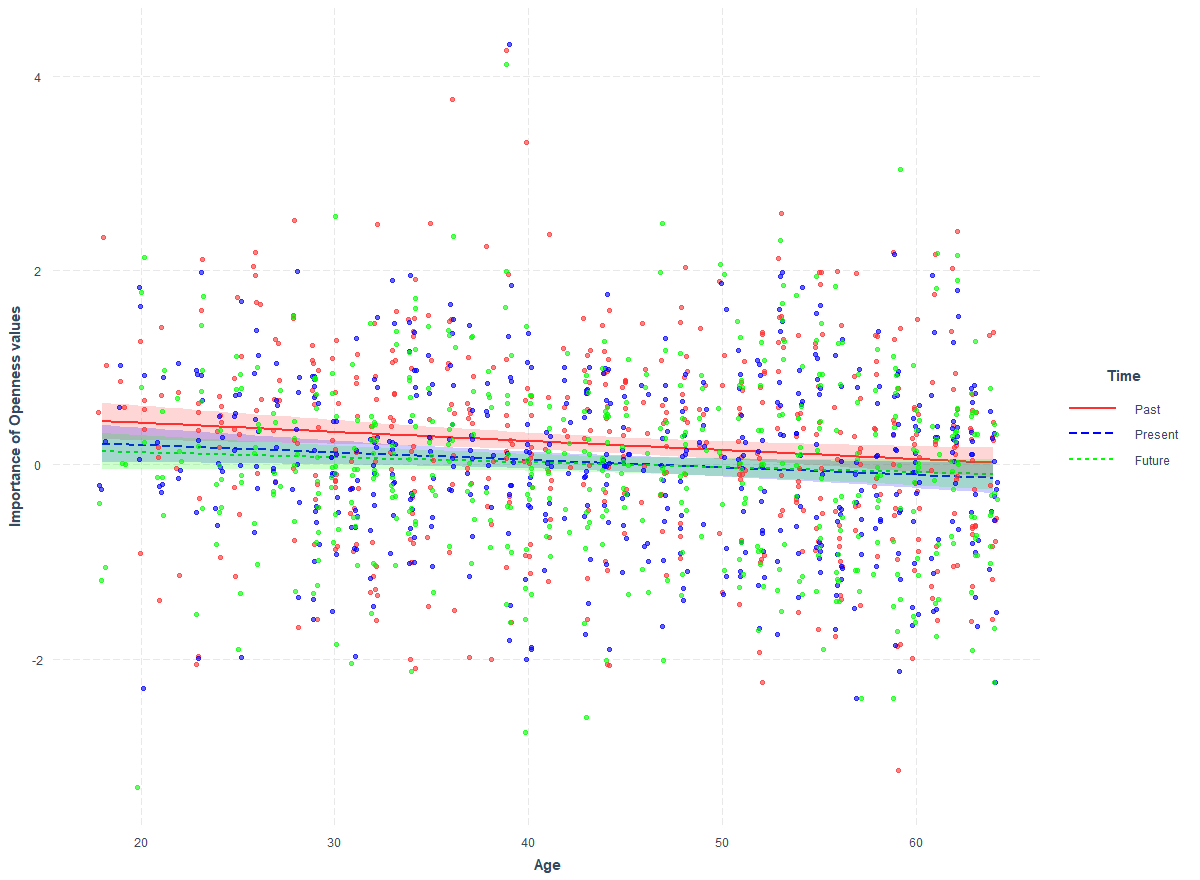


*Figure S12*. Interaction plot of age predicting centred openness values with time as a moderator.


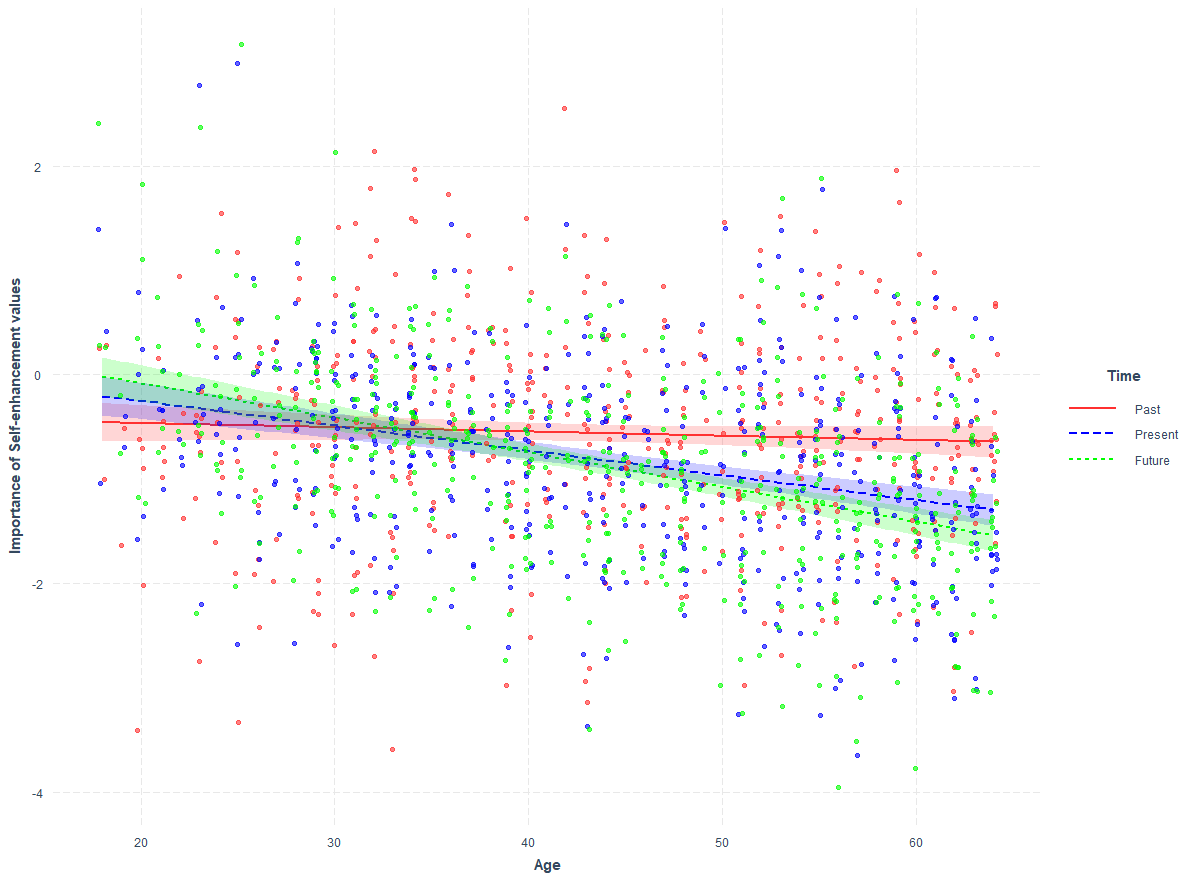


*Figure S13*. Interaction plot of age predicting centred self-enhancement values with time as a moderator.


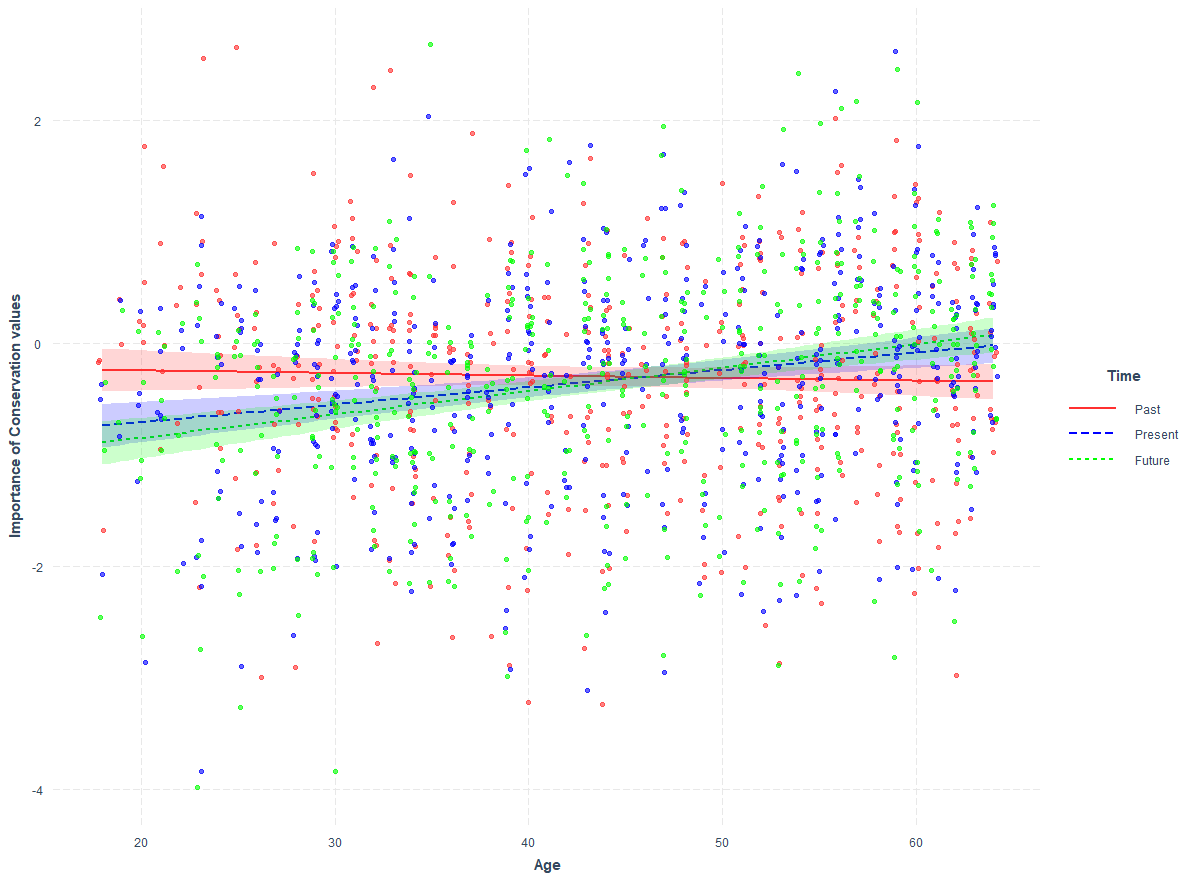


*Figure S14*. Interaction plot of age predicting centred conservation values with time as a moderator.
